# Metabolic Imbalance Triggers Adaptive Remodeling to Accelerate Diploidization in Murine Haploid Embryonic Stem Cells

**DOI:** 10.1002/advs.202522570

**Published:** 2026-04-22

**Authors:** Yi Fu, Wenhao Zhang, Yifan Zhang, Yu He, Yi Du, Yiding Zhao, Chunmeng Yao, Shengyi Sun, Xiaoyan Sheng, Qian Gao, Chao Tong, Ling Shuai

**Affiliations:** ^1^ State Key Laboratory of Medicinal Chemical Biology College of Pharmacy Nankai University Animal Resources Center and Reproductive Regulation and Institute of Transplantation Medicine Nankai University Tianjin China; ^2^ Department of Neonatology Children's Hospital of Chongqing Medical University National Clinical Research Center for Child Health and Disorders Ministry of Education Key Laboratory of Child Development and Disorders China International Science and Technology Cooperation Base of Child Development and Critical Disorders Chongqing Key Laboratory of Child Rare Diseases in Infection and Immunity Chongqing China; ^3^ Department of Obstetrics and Gynecology Chongqing Key Laboratory of Maternal and Fetal Medicine The First Affiliated Hospital of Chongqing Medical University Chongqing China

**Keywords:** diploidization, haESCs, mitochondria, mouse, optimized medium, TCA cycle

## Abstract

Murine haploid embryonic stem cells (haESCs) are ideal tools for functional genetics analyses because of their single‐genome stem cell features. However, self‐diploidization severely restricts their broader application. Although numerous attempts have been made to prevent diploidization, an effective and reliable strategy is lacking. In this study, we performed multiomics comparative analyses between haESCs and their diploidized counterparts (Di‐haESCs), which revealed that metabolic remodeling induced the adaptive evolution of haESCs toward a diploid state. Notably, an overload of intramitochondrial ROS in haESCs impaired mitochondrial bioenergetics, increasing their susceptibility to cell death and driving the progressive accumulation of diploidized cells in culture. We further found that a disrupted pyruvate–lactate balance in haESCs led to altered tricarboxylic acid (TCA) cycle activity, which was closely linked to mitochondrial dysfunction and haploid instability. Leveraging the recovery of mitochondrial function and a doubled mitochondrial number after diploidization, we performed a genome‐wide screening to identify key mitochondrial quality control (MQC) genes involved in this process. On the basis of these mechanistic insights, we developed a metabolically optimized medium for haploidy maintenance. These findings benefit haploid stem cell‐based genetic screening analyses and deepen the understanding of MQC in mammalian cells.

## Graphical abstract

In this article, Shuai and colleagues demonstrate that metabolic remodeling drives self‐diploidization in murine haploid ESCs (haESCs). Mitochondrial dysfunction and imbalanced pyruvate metabolism underlie this process. Genome‐wide screening using haESCs identifies key mitochondrial quality‐control related genes, enabling a metabolism‐based medium that maintains haploidy and expands its utility in functional genetic screening.

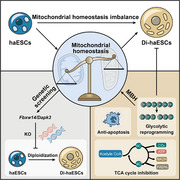

## 1 Introduction

Haploid embryonic stem cells (haESCs) derived from uniparental haploid blastocysts have been generated from many mammalian species [1, 2, 3, 4]. These self‐renewing and hemizygous haESCs have been widely used in many genetic screening approaches, including for assessment of pluripotency exiting [5], discovery of drug targets [2, 6], and investigations of bone development [7] and X‐chromosome inactivation [8]. Nevertheless, haESCs undergo rapid self‐diploidization in both daily culture and differentiation, which hinders their application in more universal ways [9]. Although they can be enriched by periodic flow cytometry sorting, it is a very time‐consuming and costly experimental strategy that is not widely accepted [10]. For the effective prevention of self‐diploidization of haESCs, the exact underlying mechanisms need to be determined. Many groups have obtained different insights into the mechanism underlying this process, including aberrant cell cycle activity [11], mitotic slippage [12], apoptosis [13, 14], and metabolic transition [15]. On the basis of the above findings, most groups chose gene editing to stabilize haploidy, which causes either genome instability or undesired genomic changes. Thus, how to sustain haploidy in a safer and more defined way is still an urgent need.

Mitochondria function as specialized metabolic hubs and signaling organelles, play a central role in cellular metabolic processes [16]. However, mitochondrial activity must be dynamically modulated via the mitochondrial quality control (MQC) system to meet various cellular demands across tissues, which encompasses ubiquitin‒proteasome degradation, mitophagy, and mito‐proteases [17]. Critically, impaired mitochondrial function not only compromises cellular viability but also activates MQC pathways [17]. Thus, it establishes a feedback loop that induces transcriptomic and proteomic remodeling [17]. Notably, extensive studies have confirmed that mitochondrial function plays an essential role in sustaining mammalian stemness and enabling cellular reprogramming [18, 19, 20, 21]. While energy metabolism and mitochondrial dynamics are known modulators of pluripotency, their influences on diploidization remain unexplored. Notably, although hypoxia promotes haploidy maintenance in human haESCs [15] and metabolic shifts occur during the diploidization of murine haESCs [20], no comprehensive study has systematically investigated metabolic processes throughout diploidization. Consequently, this represents a critical research gap, and investigation into this area may identify key regulators of diploidization and establish novel platforms for studying MQC.

In this study, we first systematically compared the transcriptome and metabolome profiles between wild‐type (WT)‐haESCs and their diploidized counterparts (Di‐haESCs) to identify key metabolic events involved in diploidization. Furthermore, we comprehensively assessed mitochondrial function throughout the diploidization process. Next, we performed genetic screening to target the MQC system and developed an optimized culture medium to ensure haploidy maintenance. Our findings not only provide a strategy for maintaining haploidy but also offer a specialized cellular model and platform for studying the MQC system.

## 2 Results

### 2.1 Stable‐haESCs (*p53*‐KO) Exhibit Low Metabolic Activities Compared with Those of WT‐haESCs

**FIGURE 1:**
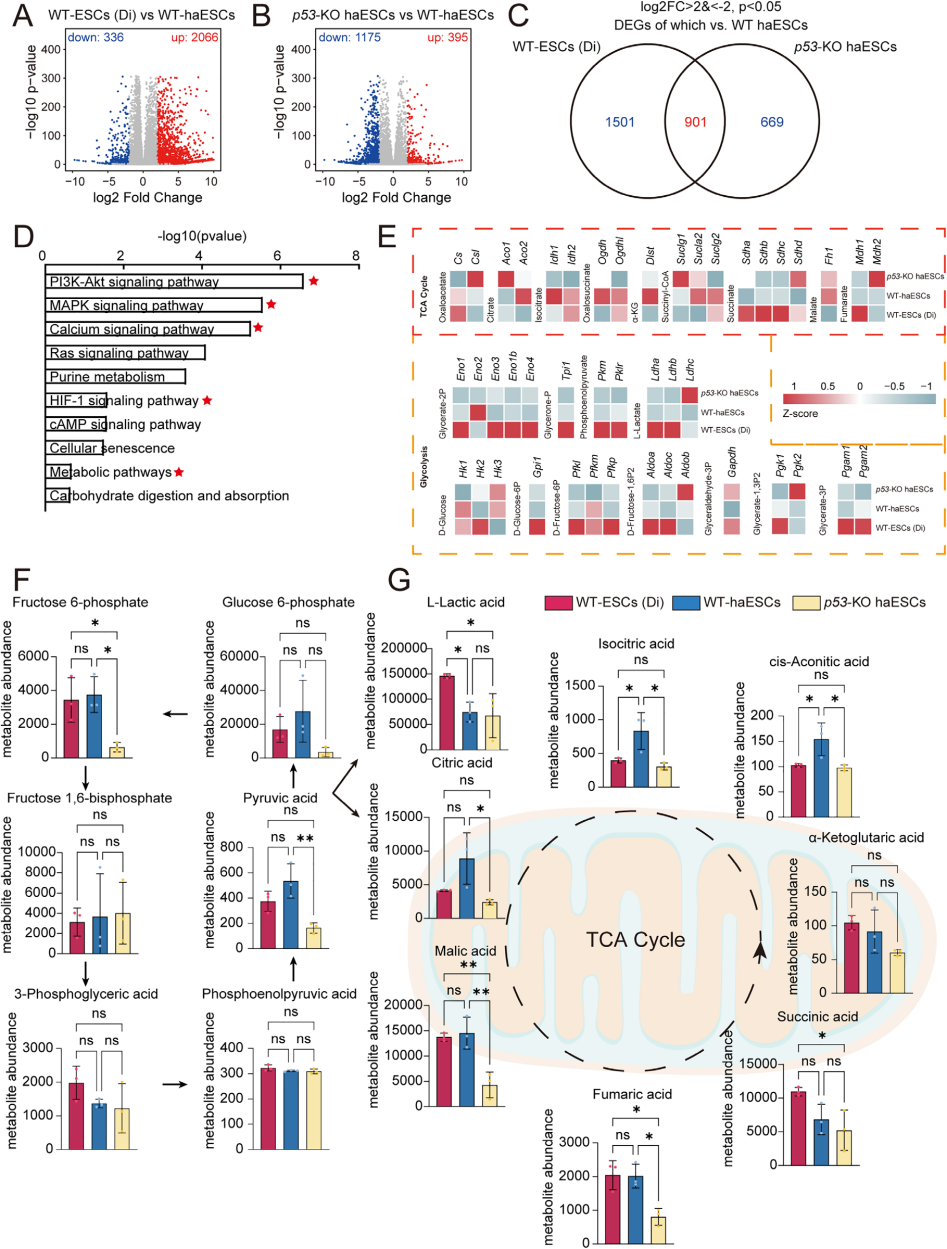
Comparison of metabolic pathways among WT‐ESCs (Di), WT‐haESCs, and *p53*‐KO haESCs. (A) Volcano plot analysis showing the DEGs between WT‐ESCs (Di) and WT‐haESCs. (B) Volcano plot analysis showing the DEGs between *p53*‐KO haESCs and WT‐haESCs. (C) Overlapping genes (901) among the genes with downregulated expression identified in WT‐haESCs (vs. WT‐ESCs (Di)) and the genes with downregulated expression identified in *p53*‐KO haESCs (vs. WT‐haESCs). (D) KEGG analysis of the above 901 overlapping genes. (E) Heatmap results showing the expression of genes involved in glycolysis and the TCA cycle in WT‐ESCs (Di), WT‐haESCs, and *p53*‐KO haESCs. (F) Bar charts displaying glycolytic metabolite abundance in WT‐ESCs (Di), WT‐haESCs, and *p53*‐KO haESCs (*n* = 3, mean ± SD). Statistical significance was determined by one‐way ANOVA with Tukey's post hoc test. *ns*, not significant; *P < 0.05; **P < 0.01. (G) Bar charts displaying TCA cycle metabolite abundance in WT‐ESCs (Di), WT‐haESCs, and *p53*‐KO haESCs (*n* = 3, mean ± SD). Statistical significance was determined by one‐way ANOVA with Tukey's post hoc test. *ns*, not significant; ^*^ *P* < 0.05; ^**^ *P* < 0.01.

Although mouse WT‐haESCs possess cellular properties and differentiation capabilities similar to those of diploid WT‐ESCs (WT‐ESCs (Di)) [1, 22], their transcriptomes differ to some degree [23]. To investigate the mechanism underlying the self‐diploidization of WT‐haESCs, we first compared the transcriptomes of WT‐ESCs (Di) and WT‐haESCs. The results revealed a great number of differentially expressed genes (DEGs) between WT‐ESCs (Di) and WT‐haESCs (Figure 1A). Given the importance of metabolic regulation in haploidy maintenance [15], we also noted that compared with WT‐ESCs (Di), WT‐haESCs exhibited a significant decrease in metabolic network regulation (Figure S1A). Our previous study revealed that *p53* knockout (*p53*‐KO) could stabilize haploid maintenance [13]. We thus used *p53*‐KO haESCs as a stable haploid control to characterize molecular changes linked to stable haploidy. We observed that *p53*‐KO haESCs displayed a unique transcriptomic profile compared with those of WT‐haESCs and WT‐ESCs (Di) (Figure 1B; Figure S1B). Interestingly, *p53*‐KO haESCs also presented a decrease in metabolic pathways compared with those of WT‐haESCs (Figure S1C). To further elucidate the mechanisms underlying the repressed metabolism underlying haploidy maintenance, we performed a combined analysis of all genes differentially expressed (DEGs, log2FC >2 & < −2, p < 0.05) between WT‐ESCs (Di) and *p53*‐KO haESCs (each vs. WT‐haESCs). Venn diagram analysis revealed 901 overlapping DEGs between WT‐ESCs (Di) and WT‐haESCs and between *p53*‐KO haESCs and WT‐haESCs (Figure 1C). KEGG enrichment analysis of these overlapping DEGs revealed that they were enriched mainly in metabolic pathways and cellular metabolism‐related signaling pathways, including the PI3K–Akt signaling pathway, MAPK signaling pathway, purine metabolism, and HIF‐1 signaling pathway (Figure 1D). Notably, the calcium signaling pathway, a core regulator of mitochondrial homeostasis, was also significantly enriched (Figure 1D). Collectively, these findings indicated that metabolic regulation might be involved in haploidy maintenance.

Because energy metabolism plays a critical role in the self‐renewal of ESCs [20, 24], we examined the expression of key enzymes involved in glycolysis and the TCA cycle. Our analysis revealed that the RNA levels of multiple enzymes (hexokinase (HK), phosphofructokinase‐1 (PFK1), pyruvate kinase (PK), citrate synthase (CS), isocitrate dehydrogenase (IDH), and oxoglutarate dehydrogenase complex (OGDC)) were significantly lower in WT‐haESCs and *p53*‐KO haESCs than in WT‐ESCs (Di) (Figure 1E). To functionally assess these transcriptional changes, we performed central carbon metabolomic profiling of WT‐ESCs (Di), WT‐haESCs, and *p53*‐KO haESCs (Figure 1F,G). Principal component analysis (PCA) revealed that these cell types had distinct metabolic profiles (Figure S1D). Consistent with the RNA‐seq results, large reductions in the levels of various carbon metabolites were observed in *p53*‐KO haESCs compared with those in the other two groups (Figure S1E–G). The quantification of key intermediates further demonstrated that *p53*‐KO haESCs presented significantly lower levels of most glycolytic intermediates (including glucose‐6‐phosphate (G6P), fructose‐6‐phosphate (F6P), and pyruvate) and TCA cycle metabolites than WT‐haESCs and WT‐ESCs (Di) did (Figure 1F,G).

Taken together, these findings indicated that WT‐haESCs and *p53*‐KO haESCs presented different metabolic profiles compared with those of WT‐ESCs (Di), particularly in terms of central carbon metabolism. Furthermore, the decrease in metabolic enzymes, coupled with the reduction in metabolic intermediates and energy substrates, suggested that a globally attenuated state of energy metabolism might facilitate the maintenance of the haploid state.

### 2.2 Remodeling of the Transcriptome and Metabolome in Central Carbon Metabolism During Self‐diploidization

**FIGURE 2:**
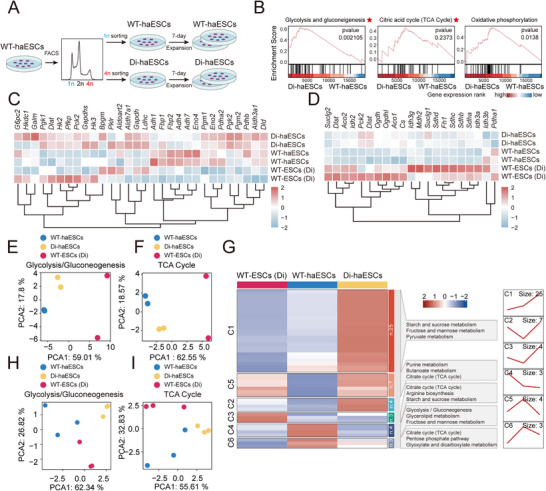
Central carbon metabolism remodeling during self‐diploidization of WT‐haESCs. (A) Schematic diagram showing the sorting and expansion strategy for WT‐haESCs (1n) and Di‐haESCs (4n). (B) GSEA of genes associated with glycolysis, the TCA cycle, and oxidative phosphorylation in a comparison of WT‐haESCs and Di‐haESCs. (C) Heatmap results showing partial expression of genes related to glycolysis in WT‐ESCs (Di), WT‐haESCs, and Di‐haESCs (*n* = 2). (D) Heatmap results showing partial expression of genes related to the TCA cycle in WT‐ESCs (Di), WT‐haESCs, and Di‐haESCs (*n* = 2). (E) Transcriptomic PCA of glycolysis/gluconeogenesis in WT‐ESCs (Di), WT‐haESCs, and Di‐haESCs (*n* = 2). The variance explained by PCA1 and PCA2 is indicated. (F) Transcriptomic PCA of the TCA cycle in WT‐ESCs (Di), WT‐haESCs, and Di‐haESCs (*n* = 2). The variance explained by PCA1 and PCA2 is indicated. (G) Heatmap results showing the mean metabolite abundance in WT‐ESCs (Di), WT‐haESCs, and Di‐haESCs (*n* = 3). (H) Metabolic PCA of glycolysis/gluconeogenesis in WT‐ESCs (Di), WT‐haESCs, and Di‐haESCs (*n* = 3). The variance explained by PCA1 and PCA2 is indicated. (I) Metabolic PCA of the TCA cycle in WT‐ESCs (Di), WT‐haESCs, and Di‐haESCs (*n* = 3). The variance explained by PCA1 and PCA2 is indicated.

Given that reduced metabolic activity was observed in stable *p53*‐KO haESCs, we hypothesized that both the transcriptome and the metabolome related to central carbon metabolism might have undergone reprogramming during self‐diploidization. To characterize these dynamics, we systematically analyzed the transcriptomes and metabolomes of WT‐haESCs, Di‐haESCs, and WT‐ESCs (Di). To ensure that the analyzed populations were pure haploid or diploid cells, we used a cell cycle and ploidy sorting strategy: we selected 1n (G0/G1 phase) cells as the WT‐haESC population and 4n (G2/M phase) cells as the Di‐haESC population, as the 2n gate would contain a mixture of G0/G1‐phase Di‐haESCs and G2/M‐phase WT‐haESCs (Figure 2A). After sorting, the cells were expanded in culture for approximately one week to obtain sufficient material for subsequent analysis and to minimize the confounding effects of cell cycle heterogeneity. DNA content analysis further confirmed the reliability of this sorting strategy, verifying the purity of the populations used in our assays (Figure S2A).

On the basis of the results of the KEGG analysis, we found that Di‐haESCs exhibited an activation of genes associated with chromosome condensation and nuclear organization, suggesting that nuclear and chromatin restructuring occurred during the diploidization of WT‐haESCs (Figure S2B). Furthermore, we observed significant activation of genes involved in glucose catabolism (glycolysis) and energy metabolism, including those involved in the TCA cycle, electron transport chain, and oxidative phosphorylation pathways, in Di‐haESCs (Figure 2B; Figure S2B). This pronounced metabolic shift was strongly supported by the elevated expression levels of related genes (Figure 2C,D). Importantly, PCA of glycolysis/gluconeogenesis and the TCA cycle indicated that Di‐haESCs were located between WT‐ESCs (Di) and WT‐haESCs (Figure 2E,F). This transcriptional convergence suggested a transition to an activated state of glycolysis and energy metabolism in Di‐haESCs. To better understand the metabolic landscape of stable haESCs, we performed an extended PCA incorporating *p53*‐KO haESCs, which revealed a distinct metabolic profile for this artificially stabilized haESC line (Figure S2C,D). Metabolomic analyses further validated these transcriptional changes, revealing a significant accumulation of intermediates in both the glycolytic cycle and the TCA cycle in Di‐haESCs relative to those in WT‐haESCs (Figure 2G). Moreover, PCA of the metabolomic profiling data revealed clear separation of Di‐haESCs from both WT‐haESCs and WT‐ESCs (Di), indicating a potential metabolic transition toward WT‐ESCs (Di) (Figure 2H,I).

Collectively, these results demonstrated that WT‐haESCs underwent comprehensive metabolic remodeling during self‐diploidization, culminating in markedly increased energy metabolism.

### 2.3 The Mitochondrial Function of WT‐haESCs is Restored after Diploidization

**FIGURE 3:**
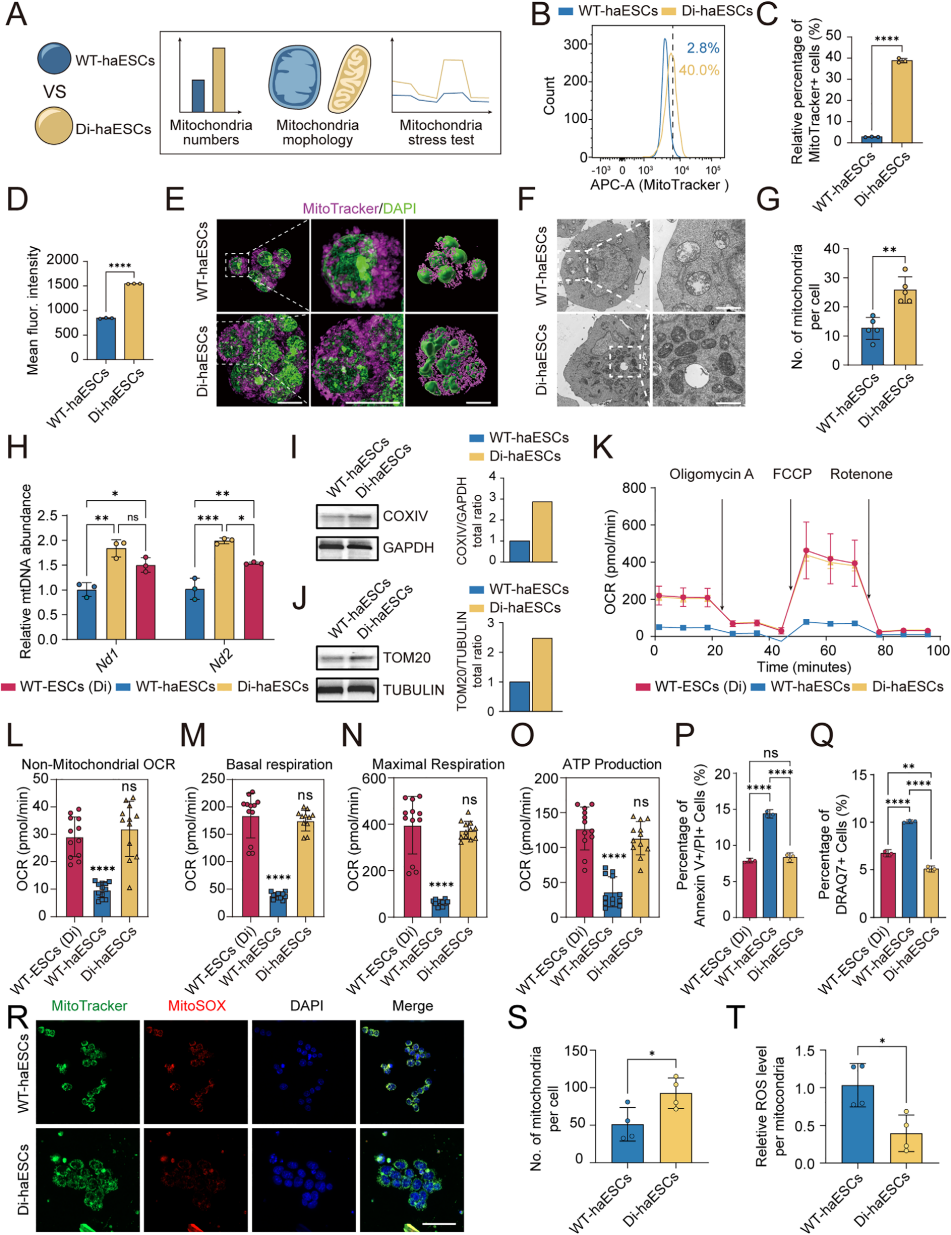
Differences in the mitochondrial state between WT‐haESCs and Di‐haESCs. (A) Schematic overview showing the comparison of mitochondria in WT‐haESCs and Di‐haESCs. (B) Representative flow cytometry analysis of the mitochondrial content of WT‐haESCs and Di‐haESCs via MitoTracker staining. (C) Quantification of the relative percentage of MitoTracker^+^ cells in WT‐haESCs and Di‐haESCs (*n* = 2, mean ± SD). Statistical significance was determined by an unpaired two‐tailed Student's *t*‐test. ^****^ *P* < 0.0001. (D) Quantification of the mean fluorescence intensity in WT‐haESCs and Di‐haESCs (*n* = 2, mean ± SD). Statistical significance was determined by an unpaired two‐tailed Student's *t*‐test. ^****^ *P* < 0.0001. (E) Representative immunofluorescence staining and Imaris 3D renderings of mitochondria in WT‐haESCs and Di‐haESCs with MitoTracker staining. Nuclei were stained with Hoechst 33342. Scale bar, 100 µm. (F) Representative TEM images of WT‐haESCs and Di‐haESCs. The images on the right show a magnified view of the structure of the mitochondria. Scale bar, 1 µm. (G) Quantitation of the number of mitochondria in WT‐haESCs and Di‐haESCs based on the TEM images (*n* = 5, mean ± SD). Statistical significance was determined by an unpaired two‐tailed Student's *t*‐test. ^**^ *P* < 0.01. (H) Quantification of relative mtDNA abundance in WT‐haESCs, Di‐haESCs, and WT‐ESCs (Di). (*n* = 3, mean ± SD). Statistical significance was determined by two‐way ANOVA followed by Tukey's post hoc test. *ns*, not significant; ^*^ *P* < 0.05; ^**^ *P* < 0.01; ^***^ *P* < 0.001. (I) Immunoblots and quantification of COXIV in WT‐haESCs and Di‐haESCs. (J) Immunoblots and quantification of TOM20 in WT‐haESCs and Di‐haESCs. (K) Extracellular flux analysis of cellular respiration and mitochondrial energy metabolism in WT‐ESCs (Di), WT‐haESCs, and Di‐haESCs (*n* = 4, mean ± SD). (L) Quantification of the nonmitochondrial OCR based on extracellular flux analysis (K) in WT‐ESCs (Di), WT‐haESCs, and Di‐haESCs (*n* = 12, mean ± SD). Statistical significance was determined by one‐way ANOVA with Tukey's post hoc test. *ns*, not significant; ****P < 0.0001. M) Quantification of basal respiration based on extracellular flux analysis (K) in WT‐ESCs (Di), WT‐haESCs, and Di‐haESCs (*n* = 12, mean ± SD). Statistical significance was determined by one‐way ANOVA with Tukey's post hoc test. *ns*, not significant; ****P < 0.0001. (N) Quantification of maximal respiration based on extracellular flux analysis (K) in WT‐ESCs (Di), WT‐haESCs, and Di‐haESCs (*n* = 12, mean ± SD). Statistical significance was determined by one‐way ANOVA with Tukey's post hoc test. *ns*, not significant; ^****^ *P* < 0.0001. (O) Quantification of ATP production based on extracellular flux analysis (K) in WT‐ESCs (Di), WT‐haESCs, and Di‐haESCs (*n* = 12, mean ± SD). Statistical significance was determined by one‐way ANOVA with Tukey's post hoc test. *ns*, not significant; ^****^ *P* < 0.0001. (P) Quantification of annexin V^+^/PI^+^ in WT‐ESCs, WT‐haESCs, and Di‐haESCs (*n* = 3, mean ± SD). Statistical significance was determined by one‐way ANOVA with Tukey's post hoc test. *ns*, not significant; ^****^ *P* < 0.0001. (Q) Quantification of DRAQ7^+^ in WT‐ESCs (Di), WT‐haESCs, and Di‐haESCs (*n* = 3, mean ± SD). Statistical significance was determined by one‐way ANOVA with Tukey's post hoc test. *ns*, not significant; ^**^ *P* < 0.01; ^****^ *P* < 0.0001. (R) Representative immunofluorescence staining of WT‐haESCs and Di‐haESCs with MitoTracker and MitoSOX staining. Nuclei were stained with Hoechst 33342. Scale bar, 100 µm. (S) Quantitation of the number of mitochondria in WT‐haESCs and Di‐haESCs based on immunofluorescent staining images (*n* = 4, mean ± SD). Statistical significance was determined by an unpaired two‐tailed Student's *t*‐test. *P < 0.05. (T) Quantification of relative ROS levels per mitochondria in WT‐haESCs and Di‐haESCs (*n* = 4, mean ± SD). Statistical significance was determined by an unpaired two‐tailed Student's *t*‐test. ^*^ *P* < 0.05.

Dysregulation of cellular energy dynamics is always accompanied by functional mitochondrial changes [25, 26]. To characterize the mitochondrial dynamics of WT‐haESCs during diploidization, we harvested WT‐haESCs and Di‐haESCs to analyze their mitochondrial number, morphology, and function (Figure 3A). We observed that Di‐haESCs exhibited significantly greater MitoTracker signaling than WT‐haESCs did, suggesting that Di‐haESCs had a greater number of mitochondria (Figure 3B,C). Quantitative analysis further confirmed that the mitochondrial volume of Di‐haESCs was approximately twice that of WT‐haESCs (Figure 3D). MitoTracker immunofluorescence staining clearly indicated that Di‐haESCs indeed had more mitochondria than WT‐haESCs did (Figure 3E). Notably, the morphology of the mitochondria exhibited a distinct shift during diploidization: WT‐haESCs predominantly contained ring‐shaped mitochondria, whereas Di‐haESCs displayed mitochondrial networks indicative of a healthier, interconnected state. Furthermore, transmission electron microscopy (TEM) revealed that the mitochondria in WT‐haESCs were swollen with faint cristae, whereas those in Di‐haESCs were slender and exhibited prominent cristae (Figure 3F). TEM quantification of mitochondria and quantitative analysis of mitochondrial abundance further confirmed that the number of mitochondria in Di‐haESCs was double that in WT‐haESCs (Figure 3G–J). The mtDNA quantification in another haploid cell line also revealed mitochondrial doubling after diploidization (Figure S3A). This shift implied potential mitochondrial damage in the haploid state, which was subsequently remodeled into a morphology with improved function during diploidization, likely driven by the elevated energy demands of Di‐haESCs.

To directly assess the function of mitochondria, we performed mitochondrial stress tests to measure the oxygen consumption rate (OCR) in WT‐ESCs (Di), WT‐haESCs, and Di‐haESCs. Di‐haESCs showed markedly significant increases in basal respiration, maximal respiratory capacity, and ATP production compared with those of WT‐haESCs (Figure 3K–O). This functional increase aligned with the transcriptional upregulation of oxidative phosphorylation factors identified by GSEA (Figure 2B). Intriguingly, the mitochondrial respiratory profile of Di‐haESCs closely resembled that of WT‐ESCs (Di), mirroring previous metabolic pathway similarities (Figure 3K–O). Critically, this enhanced mitochondrial function corresponded to a significant increase in cellular viability, as assessed by CCK‐8 assays during diploidization (Figure S3B). On the other hand, our live‐cell imaging results revealed that failed mitosis resulted in mitochondrial doubling (Figure S3C,D). This finding indicates that metabolic abnormalities are not a cause of diploidization but rather a consequence of it.

Taken together, these data demonstrated that diploidization triggered not only an increase in mitochondrial numbers but also functional reprogramming of mitochondria, culminating in marked morphological remodeling and functional enhancement.

### 2.4 Culture‐Induced Stress Promotes Adaptive Evolution of the Haploid‐to‐Diploid Transition

An imbalance in mitochondrial homeostasis is frequently associated with human diseases and aging [27]. To investigate the consequences of abnormal mitochondrial status, we explored whether mitochondrial stress induced cell death or cellular vulnerability in haploid cells. Analyses of DRAQ7 and annexin V/PI staining revealed that WT‐haESCs exhibited a higher level of apoptosis than WT‐ESCs (Di) and Di‐haESCs did (Figure 3P,Q; Figure S3E,F). Additionally, WT‐haESCs exhibited increased susceptibility to staurosporine, an apoptosis inducer (Figure S3G). These results indicated that WT‐haESCs were more fragile and more prone to apoptosis than Di‐haESCs were.

We wanted to explore the underlying mechanism of this increased apoptosis and hypothesized that impaired mitochondrial function led to the accumulation of mitochondrial reactive oxygen species (ROS), which in turn induced cell death. Given the mitochondrial dysfunction in WT‐haESCs, we performed co‐staining with MitoSOX (a probe targeting mitochondrial ROS) and MitoTracker, followed by quantitative analysis to determine both the number of mitochondria per cell and the level of ROS per individual mitochondrion (Figure 3R–T). Consistent with our previous observations, the number of mitochondria per cell was double in Di‐haESCs compared with that in WT‐haESCs (Figure 3S). Importantly, the level of ROS per mitochondrion was significantly greater in WT‐haESCs than in Di‐haESCs, directly confirming that WT‐haESCs have a greater ROS burden (Figure 3T).

On the basis of the above results and mitochondrial doubling induced by aberrant mitosis, we propose that the elevated ROS load per mitochondrion underlies the increased fragility and apoptotic susceptibility of WT‐haESCs. Aberrant mitosis acts as the initial trigger of haploid‐to‐diploid conversion, whereas mitochondrial duplication, functional enhancement, and metabolic remodeling represent secondary consequences of diploidization. This metabolic adaptation endows Di‐haESCs with greater viability and stress tolerance, enabling their gradual dominance during long‐term culture.

### 2.5 Accelerated Tricarboxylic Acid Cycle Drives Significant Diploidization

**FIGURE 4:**
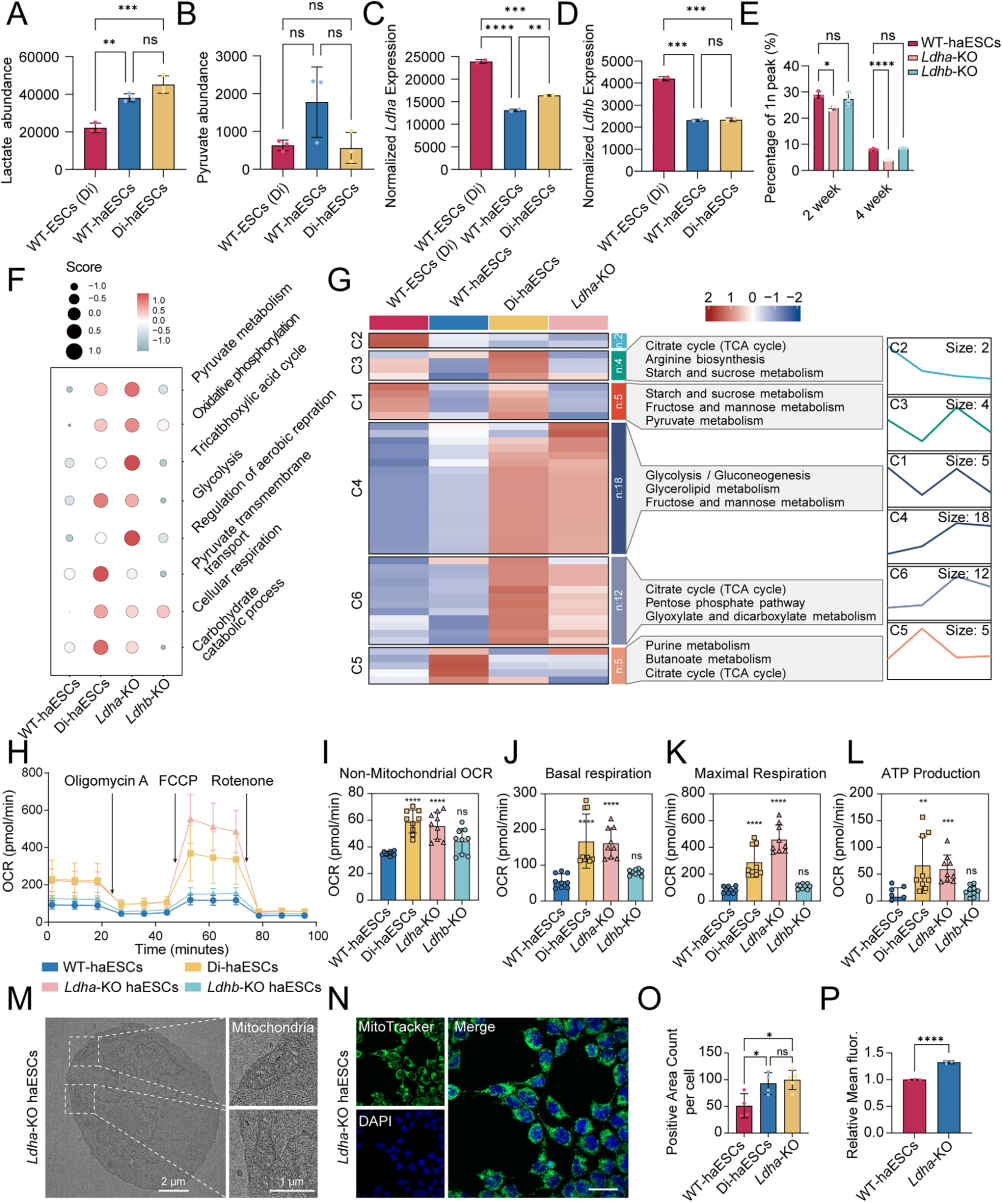
Accelerated TCA cycle induces significant diploidization. (A) The abundance of lactate in WT‐ESCs (Di), WT‐haESCs, and Di‐haESCs (*n* = 3, mean ± SD). Statistical significance was determined by one‐way ANOVA with Tukey's post hoc test. *ns*, not significant; ^**^ *P* < 0.01; ^***^ *P* < 0.001. (B) The abundance of pyruvate in WT‐ESCs (Di), WT‐haESCs, and Di‐haESCs (*n* = 3, mean ± SD). Statistical significance was determined by one‐way ANOVA with Tukey's post hoc test. *ns*, not significant. (C) Relative expression of *Ldha* in WT‐ESCs (Di), WT‐haESCs, and Di‐haESCs (*n* = 3, mean ± SD). Statistical significance was determined by one‐way ANOVA with Tukey's post hoc test. **P < 0.01; ^***^ *P* < 0.001; ^****^ *P* < 0.0001. (D) Relative expression of *Ldhb* in WT‐ESCs (Di), WT‐haESCs, and Di‐haESCs (*n* = 3, mean ± SD). Statistical significance was determined by one‐way ANOVA with Tukey's post hoc test. *ns*, not significant; ^***^ *P* < 0.001. (E) Quantification of the percentage of 1n peaks in WT‐haESCs, *Ldha*‐KO haESCs, and *Ldhb*‐KO haESCs after 2 weeks and 4 weeks of culture (*n* = 3, mean ± SD). Statistical significance was determined by one‐way ANOVA with Tukey's post hoc test. *ns*, not significant; ^*^ *P* < 0.05; ^****^ *P* < 0.0001. (F) GSVA showing energy metabolism‐related pathways in WT‐haESCs, Di‐haESCs, *Ldha*‐KO haESCs, and *Ldhb*‐KO haESCs. (G) Heatmap results showing the mean metabolite abundance in WT‐ESCs (Di), WT‐haESCs, Di‐haESCs, and *Ldha*‐KO haESCs (*n* = 3). (H) Extracellular flux analysis of cellular respiration and mitochondrial energy metabolism in WT‐haESCs, Di‐haESCs, *Ldha*‐KO haESCs, and *Ldhb*‐KO haESCs (*n* = 3, mean ± SD). (I) Quantification of the nonmitochondrial OCR based on extracellular flux analysis (H) in WT‐haESCs, Di‐haESCs, *Ldha*‐KO haESCs, and *Ldhb*‐KO haESCs (*n* = 9, mean ± SD). Statistical significance was determined by one‐way ANOVA with Tukey's post hoc test. *ns*, not significant; ^****^ *P* < 0.0001. (J) Quantification of basal respiration based on extracellular flux analysis (H) in WT‐haESCs, Di‐haESCs, *Ldha*‐KO haESCs, and *Ldhb*‐KO haESCs (*n* = 9, mean ± SD). Statistical significance was determined by one‐way ANOVA with Tukey's post hoc test. *ns*, not significant; ^****^ *P* < 0.0001. (K) Quantification of maximal respiration based on extracellular flux analysis (H) in WT‐haESCs, Di‐haESCs, *Ldha*‐KO haESCs, and *Ldhb*‐KO haESCs (*n* = 9, mean ± SD). Statistical significance was determined by one‐way ANOVA with Tukey's post hoc test. *ns*, not significant; ^****^ *P* < 0.0001. (L) Quantification of ATP production on the basis of extracellular flux analysis (H) in WT‐haESCs, Di‐haESCs, *Ldha*‐KO haESCs, and *Ldhb*‐KO haESCs (*n* = 9, mean ± SD). Statistical significance was determined by one‐way ANOVA with Tukey's post hoc test. *ns*, not significant; ^**^ *P* < 0.01; ^***^ *P* < 0.001. (M) Representative TEM images of *Ldha*‐KO haESCs. The images on the right show a magnified view of the structure of the mitochondria. Scale bars, 2 µm (left) and 1 µm (right). (N) Representative immunofluorescence staining of *Ldha*‐KO haESCs with MitoTracker staining. Nuclei were stained with Hoechst 33342. Scale bar, 100 µm. (O) Quantitation of the number of mitochondria in WT‐haESCs, Di‐haESCs, and *Ldha*‐KO haESCs based on immunofluorescence images (*n* = 4, 4, and 5; mean ± SD, respectively). Statistical significance was determined by one‐way ANOVA with Tukey's post hoc test. *ns*, not significant; *P < 0.05. (P) Quantification of the mean relative MitoTracker fluorescence intensity per cell in WT‐haESCs and *Ldha*‐KO haESCs (*n* = 3, mean ± SD). Statistical significance was determined by an unpaired two‐tailed Student's *t*‐test. ^****^ *P* < 0.0001.

Given that the activity of both the tricarboxylic acid cycle (TCA cycle) and glycolysis increased in Di‐haESCs, we noted increased lactate abundance and decreased pyruvate abundance in these cells (Figure 4A,B). Moreover, the expression of *Ldha* increased in Di‐haESCs, whereas the expression of *Ldhb* remained unchanged (Figure 4C,D). These findings suggested that disruption of pyruvate–lactate interconversion might facilitate diploidization. Lactate dehydrogenase (LDH) has five key isoforms, among which LDHA and LDHB are critically involved in the interconversion between pyruvate and lactate [28] (Figure S4A,B). Thus, we generated *Ldha*‐knockout (*Ldha*‐KO) and *Ldhb*‐knockout (*Ldhb*‐KO) haESCs through gene editing and found that their pluripotency was not affected (Figure S4C–E). After 4 weeks of culture, the percentage of 1n peak cells in *Ldha*‐KO haESCs was significantly lower than that of WT‐haESCs at week 2 and week 4 (Figure 4E). Rescue experiments further validated the specificity of LDHA (Figure S4F–H). In contrast, the percentage of 1n peak cells in *Ldhb*‐KO haESCs was not significantly different from that of WT‐haESCs (Figure 4E). This finding indicated a specific role for LDHA in maintaining the haploid state. Transcriptomic analysis corroborated the above findings (Figure 2B), with the GSVA results revealing significantly increased activity of genes related to glycolysis, the TCA cycle, and oxidative phosphorylation in Di‐haESCs compared with those in WT‐haESCs (Figure 4F). Interestingly, *Ldha*‐KO haESCs also exhibited increased activity of these pathways, suggesting that metabolic reprogramming occurred after *Ldha*‐KO and that energy metabolism pathways potentially play a role in diploidization (Figure 4F). Notably, pyruvate transmembrane transport was activated in Di‐haESCs, which might partly explain the reduction in pyruvate abundance (Figure 4F). Conversely, *Ldhb*‐KO haESCs exhibited no significant increase in these metabolic pathways or pyruvate transport activity compared with those of WT‐haESCs (Figure 4F).

To validate this reprogramming process, we also conducted metabolomic analysis and mitochondrial function analyses. Metabolomics confirmed the accumulation of glycolytic and TCA cycle intermediates in *Ldha*‐KO haESCs, mirroring that in Di‐haESCs (Figure 4G). Seahorse extracellular flux analysis further demonstrated closely aligned mitochondrial respiratory profiles between *Ldha*‐KO and Di‐haESCs (Figure 4H–L). In addition, cell viability increased after *Ldha*‐KO, which is consistent with that observed in Di‐haESCs (Figure S4I). Intriguingly, *Ldha*‐KO haESCs presented improved mitochondrial morphology and increased mitochondrial abundance, further confirming the restoration of mitochondrial function (Figure 4M–P).

Given the increased TCA cycle activity in both Di‐haESCs and *Ldha*‐KO haESCs, we hypothesize that pyruvate is critical for the process of diploidization. Notably, *Ldha*‐KO markedly increased intracellular pyruvate levels (Figure S4J), expanding the intracellular TCA substrate pool and thereby accelerating the TCA cycle. Supplementation with exogenous pyruvate accelerated diploidization in WT‐haESCs (Figure S4K), which aligned with the upregulated expression of factors associated with pyruvate transport pathways in the transcriptome. In contrast, exogenous lactate addition had no effect on diploidization (Figure S4L). Thus, pyruvate likely exerts its regulatory effect on diploidization not via conversion to lactate but potentially through fuelling the TCA cycle and affecting downstream metabolic signaling. Collectively, these data indicated that an accelerated TCA cycle drove significant diploidization.

### 2.6 Genetic Screening Identifies Mitochondrial Quality Control Modules Regulating the Diploidization of haESCs

**FIGURE 5:**
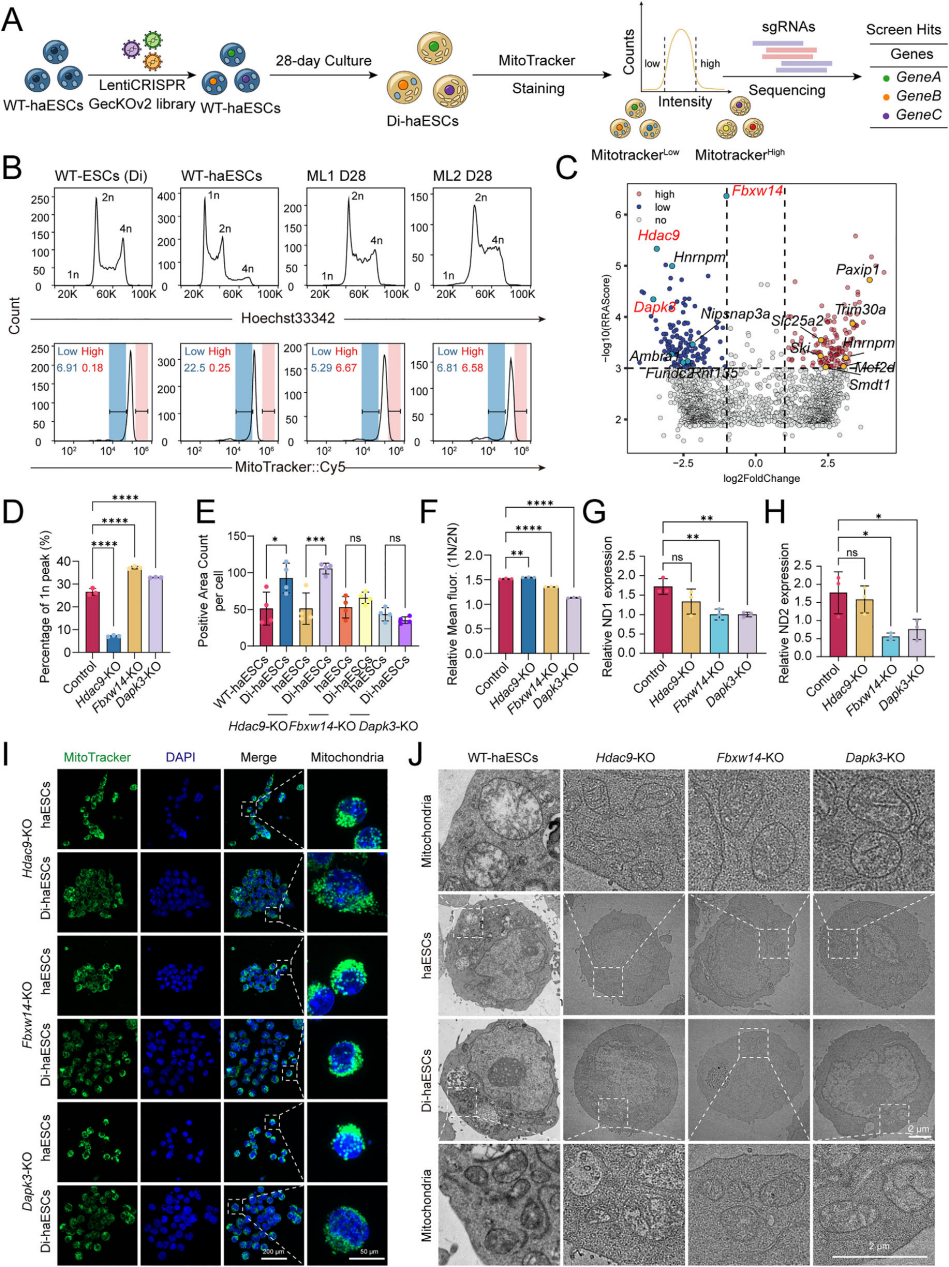
Identifying key genes involved in mitochondrial quality control during the diploidization of WT‐haESCs. (A) Schematic overview of the genome‐scale screening using WT‐haESCs for assessing mitochondrial quality during diploidization. (B) Representative flow cytometry analysis of the mitochondrial content and DNA content of WT‐ESCs (Di), WT‐haESCs, ML1 D28, and ML2 D28 with Hoechst 33342 and MitoTracker staining. (C) Plot of screening results from MAGeCK RRA analysis of the MitoTracker^high^ and MitoTracker^low^ populations. The dotted line corresponds to the stringent RRA score and log2 FoldChange cutoff values of 1 × 10^−3^ and ±1, respectively. Yellow and blue dots represent a subset of previously reported genes associated with mitochondrial quality control. (D) Quantification of the percentage of 1n peak cells in control, *Hdac9*‐KO, *Fbxw14*‐KO, and *Dapk3*‐KO haESCs after 2 weeks of culture (*n* = 3, mean ± SD). Statistical significance was determined by one‐way ANOVA with Tukey's post hoc test. ^****^ *P* < 0.0001. (E) Quantification of the number of mitochondria‐positive areas in haESCs and matched Di‐haESCs from the control, *Hdac9*‐KO, *Fbxw14*‐KO, and *Dapk3*‐KO groups (*n* = 4, mean ± SD). Statistical significance was determined by one‐way ANOVA with Tukey's post hoc test. ^*^ *P* < 0.05, ^***^ *P* < 0.001. (F) Quantification of the relative mean fluorescence intensity ratio (1N/2N) of MitoTracker staining in control, *Hdac*9‐KO, *Fbxw14*‐KO, and *Dapk3*‐KO haESCs (*n* = 3, mean ± SD). Statistical significance was determined by one‐way ANOVA with Tukey's post hoc test. ^**^ *P* < 0.01, ^****^ *P* < 0.0001. (G) Quantification of relative ND1 mRNA expression in control, *Hdac9*‐KO, *Fbxw14*‐KO, and *Dapk3*‐KO haESCs (*n* = 3, mean ± SD). Statistical significance was determined by one‐way ANOVA with Tukey's post hoc test. *ns*, not significant; ^**^ *P* < 0.01. (H) Quantification of relative ND2 mRNA expression in control, *Hdac9*‐KO, *Fbxw14*‐KO, and *Dapk3*‐KO haESCs (*n* = 3, mean ± SD). Statistical significance was determined by one‐way ANOVA with Tukey's post hoc test. *ns*, not significant; *P < 0.05. (I) Representative immunofluorescence staining of haESCs and matched *Hdac9*‐KO, *Fbxw14*‐KO, and *Dapk3*‐KO Di‐haESCs with MitoTracker (green) staining. Nuclei were stained with DAPI (blue). Scale bar, 100 µm. (J) Representative transmission electron microscopy (TEM) images of haESCs and matched control, *Hdac9*‐KO, *Fbxw14*‐KO, and *Dapk3*‐KO Di‐haESCs. The bottom panel shows a magnified view of the mitochondrial ultrastructure. Scale bar, 2 µm.

We found that mitochondrial function was extensively remodeled during the diploidization of WT‐haESCs; however, the regulatory role of the MQC system in this process remains unclear. We therefore performed a genome‐wide CRISPR knockout screen using the GeCKOv2 library [29]. We monitored mitochondrial mass via MitoTracker staining and fluorescence‐activated cell sorting (Figure 5A). We infected WT‐haESCs with a lentiviral library at an MOI<0.3 to generate single‐gene mutations with ∼500× coverage (Figure S5A). After 28 days of culture, all the infected WT‐haESCs underwent complete diploidization. We sorted the top 5% and bottom 5% of MitoTracker‐positive cells on the basis of intensity for next‐generation sequencing (NGS) (Figure 5B; Figure S5B). Notably, compared with uninfected cells, mutant haploid cells presented a greater high‐MitoTracker population (Figure 5B), which supported the rationality of our screening strategy.

We achieved high sgRNA coverage and uniform distribution in two biological replicates (Figure S5C). MAGeCK analysis identified multiple MQC regulators, including the mitochondrial autophagy inducer *Ambra1* [30], mitochondrial outer membrane protein *Fundc2* [31], mitochondrial ornithine transporter 2 *Slc25a2* [32], essential regulatory subunit of the mitochondrial calcium uniporter complex (MCU) *Smdt1* [33], death‐associated protein kinase 3 *Dapk3* [34], E3 ubiquitin‐protein ligase *Rnf135* [35] and protein NipSnap homolog 3A *Nipsnap3a* [36] (Figure 5C; Figure S5D). Gene Ontology (GO) analysis revealed that the candidate genes were enriched in mitochondrial protein modification, transport, and autophagy (Figure S5E). We further analyzed genes enriched in the high and low MitoTracker populations separately. Genes in the high‐MitoTracker group were associated with glycolysis and pyruvate‐to‐lactate conversion (Figure S5F). Both excessive and completely blocked pyruvate‐to‐lactate flux accelerated diploidization (Figure 4; Figure S5F), indicating that balanced metabolic flux was essential for haploid stability. Genes in the low‐MitoTracker group were linked to mitochondrial function, apoptosis, and ROS scavenging (Figure S5F).

To validate the reliability of our genome‐wide screening results, we focused on the top candidate genes identified in the low‐MitoTracker‐stained population, in which gene KO was expected to maintain low mitochondrial abundance and promote haploid stability (Figure 5C). We selected three key candidate genes (*Hdac9*, *Fbxw14*, and *Dapk3*) for functional validation. Our results revealed that *Fbxw14*‐ and *Dapk3*‐KO effectively stabilized haploidy and maintained low mitochondrial numbers even after diploidization (Figure 5D–I), with TEM observations confirming that the mitochondria in these two KO lines retained abnormal, unhealthy morphology after diploidization (Figure 5J). In contrast, *Hdac9*‐KO accelerated diploidization without significant changes in mitochondrial number, and mitochondrial morphology recovered normally after diploidization (Figure 5D–J). These functional validation data confirmed the effectiveness of our screening approach.

Taken together, these data indicated that mitochondrial remodeling during the diploidization process involved MQC, providing guidance for the development of WT‐haESC‐compatible culture medium and a robust screening model for MQC research.

### 2.7 Inhibition of Mitochondrial Pyruvate Uptake Stabilizes Haploid Cell Cultures

Previous strategies for maintaining haploidy have relied predominantly on genetic manipulation, but these genomic perturbations limit the broader application of WT‐haESCs. The development of a culture medium specifically conducive to haploidy maintenance is therefore necessary for the establishment of efficient haploid genetic screening platforms. Our genetic screening results revealed that the balance of pyruvate‐to‐lactate conversion served as a key determinant of haploid stability. We also found that *Ldha*‐KO accelerates diploidization by altering pyruvate metabolism and downstream metabolic homeostasis. Given that increased mitochondrial function and an accelerated TCA cycle drove diploidization, we hypothesized that inhibiting the TCA cycle could stabilize the haploid state (Figures 3, 4, 5). We therefore added several energy metabolism pathway inhibitors, including 2‐deoxy‐D‐glucose (2‐DG, a glycolysis inhibitor [37]), devimistat (a mitochondrial metabolic inhibitor [38]), and UK5099 (a mitochondrial pyruvate transporter inhibitor [39]), to the WT‐haESC medium.

**FIGURE 6:**
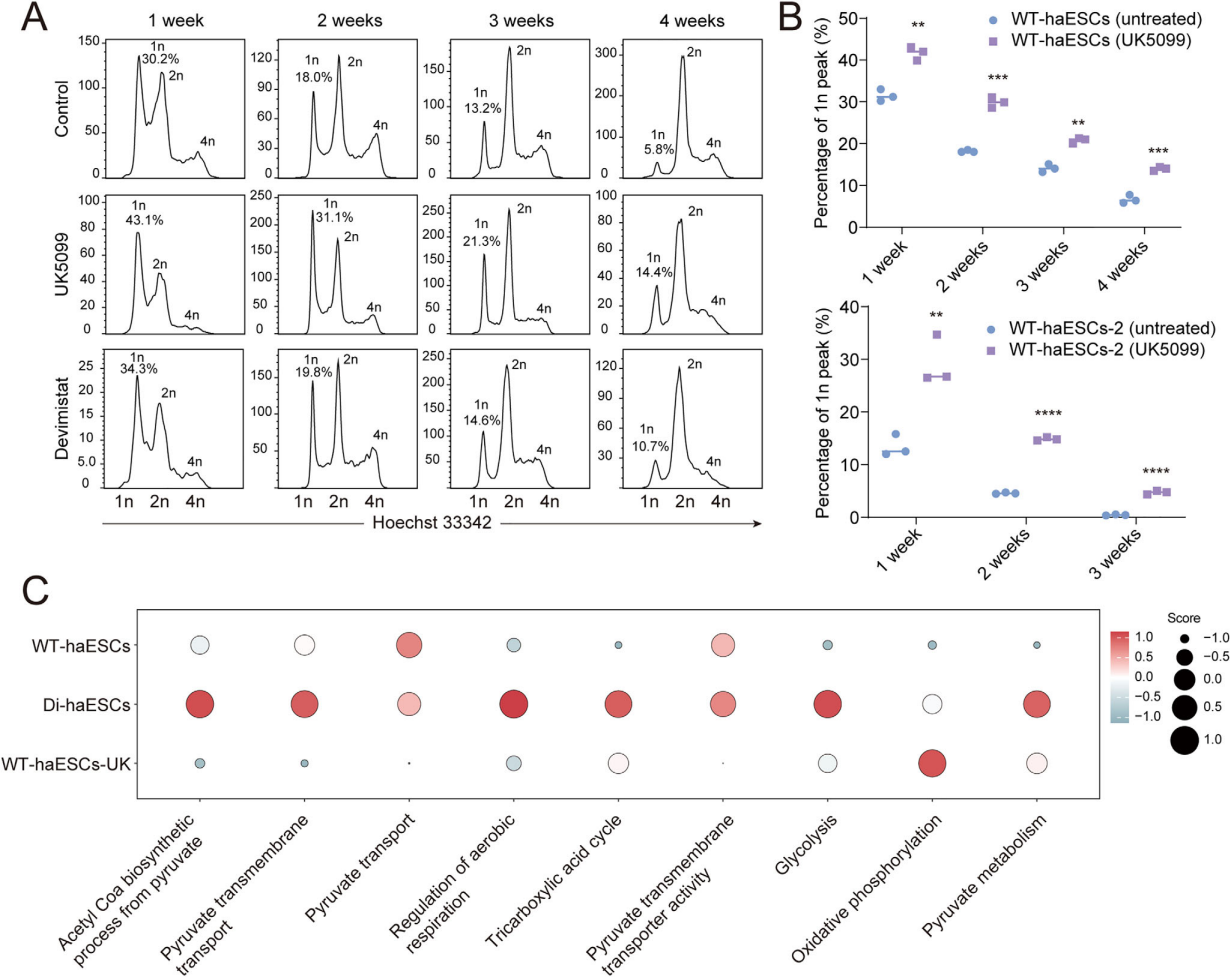
Inhibition of mitochondrial pyruvate uptake guarantees haploidy maintenance. (A) Representative FACS analysis of DNA content in WT‐haESCs cultured in 2iL medium with or without UK5099 and devimistat for 1–4 weeks. (B) Analysis of the percentages of the 1n peak in WT‐haESCs (cell line 1 and cell line 2) cultured in 2iL medium with or without UK5099 for 1–4 weeks (*n* = 3, mean ± SD). Statistical significance was determined by an unpaired two‐tailed Student's *t*‐test. ^**^ *P* < 0.0001; ^***^ *P* < 0.001; ^****^ *P* < 0.0001. (C) GSVA analysis showing energy metabolism‐related pathways in WT‐haESCs cultured in 2iL medium with or without UK5099 and in Di‐haESCs.

After 2 weeks of inhibitor treatment, both UK5099 and 2‐DG significantly improved haploid maintenance compared with that in the untreated group (Figure S6A). However, 2‐DG severely compromised the viability and proliferation of WT‐haESCs (Figure S6B,C). To evaluate long‐term efficacy, we cultured WT‐haESCs for 4 weeks with UK5099 or devimistat. The UK5099 group consistently maintained a significantly greater proportion of haploid cells than the other groups, whereas the devimistat group exhibited only a minimal effect (Figure 6A,B). This haploid‐preserving effect of UK5099 was further validated in another independent haploid cell line (Figure 6B). UK5099 treatment also did not affect the viability or pluripotency of WT‐haESCs (Figure S6D,E).

Although UK5099‐treated haESCs (WT‐haESCs‐UK) retained a general similarity to the untreated cells, we detected 609 genes with downregulated expression in WT‐haESCs‐UK (S6F,G). GSVA revealed that pyruvate transmembrane transport and mitochondrial pyruvate uptake were significantly lower in WT‐haESCs‐UK than in untreated WT‐haESCs (Figure 6C). The increased oxidative phosphorylation observed in WT‐haESCs‐UK likely reflected a compensatory response of other metabolic pathways. These data demonstrated that inhibition of pyruvate transport mitigated the haploid‐to‐diploid transition by suppressing mitochondrial TCA cycle activity.

Collectively, these results confirmed that restricting pyruvate entry into mitochondria provided an effective and nongenetic approach to sustain haploidy.

### 2.8 An Optimized Metabolism‐Based Medium for Stably Sustaining Haploidy

**FIGURE 7:**
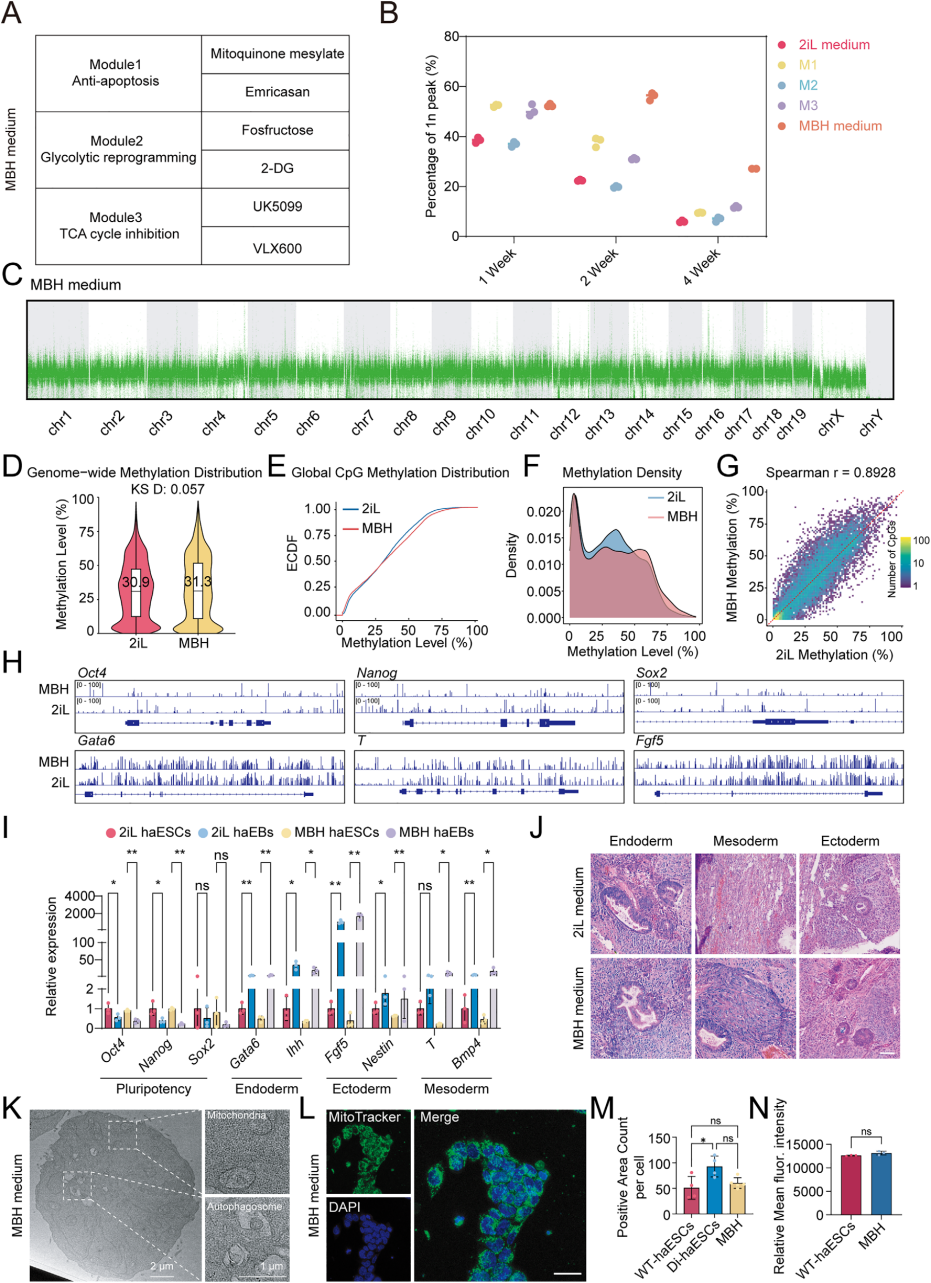
Optimized metabolism‐based culture medium for sustaining haploidy. (A) Different combinations of small molecules used for maintaining haploidy. (B) Analysis of the percentages of the 1n peak in WT‐haESCs cultured in different combinations for 1–4 weeks (*n* = 3, mean ± SD). (C) Whole‐genome CGH profile of WT‐haESCs cultured in MBH medium, showing copy number variation across chromosomes chr1 to chrY. (D) Violin plot showing the distribution of genome‐wide DNA methylation levels in 2iL medium and MBH medium‐cultured WT‐haESCs, with the Kolmogorov‒Smirnov (KS) test D value indicated. (E) Empirical cumulative distribution function (ECDF) plot of global CpG methylation distribution in 2iL medium and MBH medium‐cultured WT‐haESCs. (F) Density histogram of genome‐wide CpG methylation levels in 2iL medium and MBH medium‐cultured WT‐haESCs. (G) Spearman correlation analysis of genome‐wide CpG methylation levels in 2iL medium and MBH medium‐cultured WT‐haESCs, with a Spearman correlation coefficient of r = 0.8928 indicated. (H) Genome browser view of DNA methylation peaks at pluripotency genes (*Oct4*, *Nanog*, and *Sox2*) and lineage‐specific differentiation genes (*Gata6*, *T*, and *Fgf5*) in 2iL medium and MBH medium‐cultured WT‐haESCs. (I) Quantification of the relative mRNA expression of pluripotency markers (*Oct4*, *Nanog*, and *Sox2*), endoderm markers (*Gata6*, *Ihh*), ectoderm markers (*Fgf5* and *Nestin*), and mesoderm markers (*T* and *Bmp4*) in 2iL medium and MBH medium‐cultured WT‐haESCs, and their corresponding embryoid bodies (EBs) after 7 days of differentiation (*n* = 3, mean ± SD). Statistical significance was determined by an unpaired two‐tailed Student's *t*‐test. *ns*, not significant; ^*^ *P* < 0.05; ^**^ *P* < 0.01. (J) Representative hematoxylin and eosin (HE) staining images of teratomas derived from 2iL medium and MBH medium‐cultured WT‐haESCs, showing differentiated structures of endoderm, mesoderm, and ectoderm lineages. Scale bar, 100 µm. (K) Representative TEM images of WT‐haESCs cultured in MBH medium. The right panels show magnified views of the autophagosome and mitochondrial ultrastructure. Scale bars, 2 µm (left) and 1 µm (right). (L) Representative immunofluorescence staining of WT‐haESCs cultured in MBH medium with MitoTracker staining. Nuclei were stained with DAPI. Scale bar, 100 µm. (M) Quantitation of the number of mitochondria‐positive areas per cell in WT‐haESCs, Di‐haESCs, and MBH medium‐cultured WT‐haESCs (*n* = 4, 4, and 5, respectively; mean ± SD). Statistical significance was determined by one‐way ANOVA with Tukey's post hoc test. *ns*, not significant; *P < 0.05. (N) Quantification of the relative mean fluorescence intensity of MitoTracker staining in WT‐haESCs and MBH medium‐cultured WT‐haESCs (*n* = 3, mean ± SD). Statistical significance was determined by an unpaired two‐tailed Student's *t*‐test. *ns*, not significant.

Our genetic screening revealed that a balanced pyruvate‐to‐lactate metabolic flux was critical for the suppression of diploidization (Figure 5). Excessive mitochondrial activity (Figures 3 and 4), ROS production, and apoptosis (Figure 3) not only accelerated diploidization but also accounted for the intrinsic fragility of WT‐haESCs. Conversely, appropriately compromised mitochondrial function contributed to the stabilization of haploidy (Figures 3 and 6). On the basis of the above findings, we designed and constructed three functional modules, namely, the anti‐apoptotic module (M1, mitoquinone mesylate [40] and emricasan [41]), the glycolytic reprogramming module (M2, fructose‐1,6‐bisphosphate and 2‑DG [37]), and the TCA cycle inhibition module (M3, UK5099 [39] and VLX600 [42]). The combination of these modules was termed metabolism‑based haploid medium (MBH medium) (Figure 7A).

We first optimized the concentrations of all the compounds to avoid adverse effects on cell proliferation (Figure S7A). Guided by our genetic screening results, we reduced the concentration of 2‑DG so that it mediated glycolytic reprogramming instead of exerting global glycolytic inhibition (Figure S7B,C). Fructose‑1,6‑bisphosphate was included to replenish glycolytic intermediates and sustain core metabolic flux. Mitoquinone mesylate [40] and emricasan [41] were applied to scavenge mitochondrial ROS and inhibit pancaspase activity, whereas UK5099 [39] and VLX600 [42] were utilized to inhibit mitochondrial TCA cycle activity.

On the basis of the above optimization, we examined the haploid‐stabilizing effects of individual modules and the complete MBH medium. After 4 weeks of culture, the cells maintained in 2iL medium underwent extensive diploidization. In contrast, supplementation with M1 or M3 significantly inhibited diploidization at the early stage and exerted a moderate stabilizing effect at the late stage (Figure 7B; Figure S7D). The glycolytic reprogramming module M2 alone exhibited only a mild effect. Notably, the combined application of all three modules effectively prevented diploidization throughout the entire culture period at both the early and late stages (Figure 7B; Figure S7D).

To validate the safety and long‐term stability of WT‐haESCs cultured in MBH medium, we performed a series of comprehensive analyses, including comparative genomic hybridization (CGH), whole‐genome bisulfite sequencing (WGBS), and pluripotent differentiation assays, on cells after 10 passages. CGH detection revealed no obvious genomic copy number variation in MBH medium‐cultured haESCs, confirming their intact genome (Figure 7C). WGBS profiling revealed highly similar methylation patterns between MBH medium‐treated cells and control cells, with unchanged methylation status of key pluripotency genes and differentiation genes, indicating unperturbed epigenetic homeostasis (Figure 7D–H). In addition, MBH medium‐cultured haESCs retained normal differentiation competence to generate cells representing three germ layers efficiently (Figure 7I,J; Figure S7E,F).

Furthermore, we assessed mitochondrial characteristics in WT‐haESCs cultured in MBH medium by TEM, MitoTracker immunofluorescence staining and mitochondrial count analysis. Compared with 2iL haESCs, MBH haESCs exhibited obvious mitophagy and abnormal vacuolated mitochondrial morphology (Figure 7K,L). The results revealed no significant difference in the total number of mitochondria between the MBH haESCs and 2iL haESCs (Figure 7M,N). These observations demonstrated that MBH medium sustained the haESCs in a less healthy mitochondrial state, which was consistent with our core finding that compromised mitochondrial function supported the stability of the haploid state.

In summary, we established a chemically defined MBH medium that effectively reduces spontaneous diploidization via metabolic regulation. These findings reveal the core mechanism of metabolic reprogramming in haploidy maintenance, provide technical support for stable, genome‐unperturbed WT‐haESCs, and lay a foundation for efficient haploid genetic screening platforms.

## 3 Discussion

Mammalian haESCs are valuable for functional genetic studies when combined with genomic mutation strategies [10, 43], but their application is hindered by spontaneous diploidization and hemizygosity loss. To elucidate the underlying mechanism, we performed combined transcriptomic and metabolomic analyses and found that haESCs underwent complicated metabolic remodeling during self‐diploidization, culminating in markedly increased energy metabolism. These findings indicated that repressed glycolysis and the TCA cycle were suitable for haploidy, whereas increased TCA cycle activity and mitochondrial function drove diploidization. Furthermore, we found that increased susceptibility to stress‐induced apoptosis in WT‐haESCs drove adaptive evolution toward stress‐tolerant diploid cells. Leveraging the key role of MQC in this process, we performed a genome‐wide screen to identify key regulators, with validation confirming that *Fbxw14*‐ or *Dapk3*‐KO stabilized haploidy. Given the limitations of existing strategies to inhibit diploidization [11, 12, 13, 14, 15], we developed a nongenotoxic metabolism‐based strategy to maintain haploidy, which revealed a novel regulatory mechanism and facilitated haploid genetic screening as a powerful tool.

Notably, our study revealed that despite reduced metabolic activity in *p53*‐KO haESCs, *p53* appeared to maintain haploidy through a distinct mechanism. Multiomics analyses collectively revealed significant differences between *p53*‐KO and WT‐haESCs. This discrepancy might be largely explained by the divergent regulatory logic of these two interventions, which targeted the core regulatory axis of mitochondrial overactivation, ROS accumulation, apoptosis, and adaptive diploidization from opposing ends. Specifically, *p53*‐KO acted downstream of this axis and weakened mitochondria‐mediated apoptosis, thereby negating the apoptotic selection pressure that drove diploidization even under mitochondrial stress. However, this strategy relies on permanent genomic modification, which restricts the applicability of haESCs in subsequent genetic screening. In contrast, the MBH medium acted upstream of the core axis and directly repressed TCA cycle activity and pyruvate uptake, thus eliminating the sources of oxidative stress and apoptotic triggers at the root. Our data confirmed that MBH‐cultured haESCs retained an intact genomic stability, normal pluripotency and viability, verifying that this mitochondrial phenotype reflected a controllable metabolic state rather than irreversible cellular damage.

Our findings also provided novel insights into the metabolic regulation of genome stability in haESCs. The results of our genetic screening indicated that the enrichment of genes associated with the negative regulation of glucose catabolism to lactate via pyruvate was closely linked to rapid diploidization. This observation suggested that elevated flux from pyruvate to lactate contributed to diploidization. We further observed that direct KO of *Ldha* also accelerated diploidization even though this intervention blocked the conversion of pyruvate to lactate. Taken together, these outcomes demonstrated that both excessive activation and complete blockade of pyruvate‐to‐lactate flux disturbed metabolic homeostasis and impaired haploid stability. Moreover, our results indicated that gene KO represented an overly simplistic and absolute approach to metabolic regulation. Metabolic balance served as the key determinant, and thus, the application of small‐molecule compounds constituted a superior strategy for stabilizing haploid cells. Pharmacological interventions allowed fine‐tuning of pyruvate fate rather than complete activation or inhibition of related processes. These interventions moderately reduced pyruvate‐to‐lactate flux while maintaining appropriate metabolic flow. Such balanced regulation effectively restored cellular homeostasis and sustained haploid genome stability.

Taken together, the results of our study link metabolic regulation to chromosome ploidy maintenance, demonstrating that metabolic reprogramming drives adaptive diploidization evolution in WT‐haESCs. We also developed a nongenetic, genome editing‐free metabolic strategy for stable haploidy maintenance and established a genome‐wide screening platform for MQC studies. These findings provide an ideal tool for high‐throughput functional genetic screening and new insights into the crosstalk between cellular metabolism and genome stability.

## 4 Experimental Section

### 4.1 Cell Culture

WT‐haESCs were cultured in N2B27/2iLIF medium (2iL) on feeder cells as described previously [44]. The medium was prepared using Ndiff227 medium (Y40002; Takara, Japan) supplemented with 1 µm PD0325901 (HY‐10254; MCE, USA), 3 µM CHIR99021 (HY‐10182; MCE, USA), and 1 × 10^4^ U/mL mouse LIF (HY‐P7084; MCE, USA). Inhibitors, including UK5099 (HY‐15475; MCE, USA), 2‐DG (HY‐13966; MCE, USA), devimistat (HY‐15453; MCE, USA), mitoquinone mesylate (HY‐100116A; MCE, USA), emricasan (HY‐10396; MCE, USA), fosfructose (HY‐106950; MCE, USA), and VLX600 (HY‐12406; MCE, USA), were added to 2iL when necessary. All the cells were cultured in a humidified incubator (4111; Thermo, USA) at 37°C with 5% CO_2_ and 21% O_2_, and the medium was changed every day. All cells were routinely checked for free of mycoplasma contamination.

MBH medium was prepared by supplementing 2iL medium with 2.5 µm emricasan, 25 nm mitoquinone mesylate, 0.25 mm 2‐DG, 25 µm fosfructose, 250 nm VLX600, and 25 µm UK5099.

### 4.2 Plasmid Construction and Electroporation

To generate *Ldha*‐ and *Ldhb*‐KO haESCs, specific single‐guide RNAs (sgRNAs) were designed using the CRISPOR website (http://crispor.tefor.net) and cloned and inserted into the PX458 vector (Addgene, #48138). Approximately 8 µg of the constructed plasmid was electroporated into 1 × 10^6^ cells using an electroporator (4D‐Nucleofector System; Lonza, Switzerland). The sequences of the sgRNAs and genotyping primers are provided in Data S1.

### 4.3 Cell Sorting and Flow Cytometric Analysis

For enrichment or analysis of haploid cells, the cells were incubated with Hoechst 33342 (5 µg/mL) (H3570; Invitrogen, USA) for 20 min at 37°C and filtered through a 40‐µm cell strainer (352340; BD Biosciences, USA). Mouse WT‐ESCs (Di) were used as a diploid control. Cells were sorted or analyzed on a MoFlo sorter (Astrios EQ; Beckman, USA).

To visualize the mitochondria, the cells were incubated with 25 nm MitoTracker (A66440; Thermo, USA) for 30 min at 37°C. After being filtered through a 40‐µm cell strainer, the cells were washed three times with PBS. The fluorescence was detected using a flow cytometer (LSR Fortessa; BD Biosciences, USA), and the data were analyzed using FlowJo software (version 10.8.1).

### 4.4 Cell Viability and Apoptosis Assay

Cell viability was determined using the Cell Counting Kit‐8 (CCK‐8) following the manufacturer's protocol (40203ES76; Yeasen, China). Briefly, a total of 1 × 10^3^ cells were seeded into each well of a 96‐well plate, cultured for 3 days, and then incubated with CCK‐8 for 4 h. This was followed by absorbance measurement using a microplate reader (Spark; Tecan, Austria).

An apoptosis assay was conducted using either an Annexin V‐Alexa Fluor 488/PI Apoptosis Detection Kit (40305ES60; Yeasen, China) or DRAQ7 (MX4237; MK Bio, China) in accordance with the manufacturers’ instructions. Briefly, cells were dissociated into single‐cell suspensions and then washed twice with ice‐cold PBS. The cells were subsequently incubated with annexin V‐Alexa Fluor 488/PI or DRAQ7 at room temperature for 15 min and analyzed using a flow cytometer (LSR Fortessa; BD Biosciences, USA).

### 4.5 Mitochondrial Stress Test

The mitochondrial stress tests were performed using a Seahorse XF Cell Mitochondrial Stress Test Kit (103015‐100; Agilent, USA) according to the manufacturer's instructions. Briefly, 1 × 10^4^ cells were seeded in XF24 cell culture microplates coated with 0.2% gelatin (V900863; Sigma, USA) at 37°C overnight. The medium was changed to Seahorse XF DMEM supplemented with 10 mm glucose, 1 mm pyruvate, and 2 mm glutamine (from the kit). The cells were incubated at 37°C in a non‐CO_2_ incubator for 1 h. To test for mitochondrial stress, 1.5 µm oligomycin A, 1 µm FCCP, and 0.5 µm rotenone/antimycin A were injected into ports A, B, and C, respectively. The mitochondrial oxygen consumption rate (OCR) and extracellular acidification rate (ECAR) were measured and recorded using a Seahorse XF‐24 analyzer. The data were analyzed using Seahorse Wave software.

### 4.6 Embryoid Body Differentiation and Teratoma Formation

In vitro embryoid body (EB) differentiation and in vivo teratoma formation assays (including tissue sectioning and hematoxylin and eosin (H&E) staining) were performed following our previously published protocols [13].

### 4.7 Immunofluorescence Staining

The samples were fixed with 4% paraformaldehyde (P6148; Sigma, USA) for 30 min at room temperature. After being washed three times, the samples were permeabilized and blocked using 0.1% Triton X‐100 (T8787; Sigma, USA) diluted in 5% BSA (A1933; Sigma, USA) for 1 h at room temperature. Next, the samples were incubated overnight at 4°C with primary antibodies. Afterward, the samples were washed three times with PBS, followed by incubation with fluorescence‐conjugated secondary antibodies for 2 h at room temperature in the dark. The nuclei were stained with Hoechst 33342 for 15 min at room temperature. Mitochondria were stained using MitoTracker for 30 min before fixation. Immunofluorescence images were captured on confocal laser scanning microscopes (TCS SP8 (Leica, Germany); LSM800 (Zeiss, Germany); and Elyra 7 (Zeiss, Germany)). 3D images of the mitochondria and nuclei were generated using Imaris 9.9.0 software (Bitplane AG). The quantification of fluorescence intensity was performed using ImageJ software (version v1.54r).

The primary antibodies used for immunostaining were as follows: anti‐OCT4 (sc‐5279; Santa Cruz, USA), anti‐SOX2 (sc‐365823; Santa Cruz, USA), and anti‐NANOG (sc‐374103; Santa Cruz, USA). The secondary antibodies used for immunostaining were as follows: Alexa Fluor 647 AffiniPure Goat Anti‐Mouse IgG (H+L) (33213ES60; Yeasen, China), Alexa Fluor 647 AffiniPure Goat Anti‐Rabbit IgG (H+L) (33113ES60; Yeasen, China), FITC‐conjugated goat anti‐rabbit IgG (H+L) (AS011; ABclonal, China), and FITC‐conjugated goat anti‐mouse IgG (H+L) (AS001; ABclonal, China).

### 4.8 Cellular Metabolite Collection and LC‒MS/MS Detection

Cells were trypsinized using 0.25% Trypsin/EDTA (25200072; Thermo, USA) for 3 min, flash‐frozen, resuspended in ddH_2_O (300 µL), mechanically homogenized (bead mill, 35 Hz, 4 min), and sonicated (ice‐water bath, 15 min × 3). Aliquots (250 µL) were mixed with cold methanol (−40°C, 750 µL; vortexed for 30 s) and incubated (−40°C, 1 h), after which the residue was saved for the BCA protein assay. After centrifugation (12,000 rpm, 4°C, 15 min), the supernatant (900 µL) was concentrated, reconstituted in ddH_2_O (180 µL), filtered (0.22 µm), centrifuged, and stored in autosampler vials; a pooled QC sample was prepared. HPLC‒MS/MS was performed using a Dionex ICS‐6000 (Thermo, USA) with AG11‐HC/AS11‐HC columns (2 × 50 mm), mobile phases (A: 100 mm NaOH; B: H_2_O) with postcolumn addition (methanol/2 mm acetic acid; 0.15 mL/min), a column at 30°C, an autosampler at 4°C, and 5 µL of injection.

For mass spectrometric detection, a Q Exactive Hybrid Quadrupole‐Orbitrap mass spectrometer (Thermo, USA) in negative electrospray ionization (ESI) (‐) mode (30 arb sheath gas, 10 arb aux gas, 350°C capillary temperature, and −3.8 kV spray voltage) was used to acquire full‐scan spectra (m/z 100–1200, 70k resolution) via Xcalibur v4. Data analysis involved peak ID (Skyline v21.1), format conversion to mzXML (ProteoWizard MSConvert v3.0), and multivariate statistics (XCMS in R v4.2.1).

### 4.9 Transmission Electron Microscopy

The cells were subsequently washed twice with PBS and dissociated using 0.25% trypsin/EDTA. Following centrifugation, the cell pellets were fixed in a TEM fixative solution (G1102; Servicebio, China) at 4°C for 4 h. The samples were processed by a local company (Servicebio, China), and images were captured by cryo‐transmission electron microscopy (cryo‐TEM) (Talos L120C G2; FEI, USA).

### 4.10 Live Imaging

Live imaging was performed using a label‐free live‐cell microscopy system (SC3000; Zircon, China) with a 40× objective lens. For the analysis of MitoTracker‐stained cells, long‐term time‐lapse imaging was conducted in intensity diffraction tomography (IDT) mode, images were taken every 8 min with z stacks for more than 24 h, and image processing and phenotypic analysis were performed using the system's built‐in analysis software.

### 4.11 Quantitative Analysis of Mitochondrial Abundance

Relative mitochondrial DNA (mtDNA) abundance was assessed as previously described [21]. Briefly, the genomes of samples with the same cell count were extracted using a Universal Genomic DNA Kit (CW2298M; CWBIO, China). Quantitative PCR (qPCR) was performed using Hieff SYBR Green Master Mix (11201ES08; Yeasen, China) on a CFX96 Touch Real‐Time PCR Detection System (Bio‐Rad, USA). The primer sequences are listed in Data S1.

### 4.12 CRISPR Screening and Data Analysis

Mouse CRISPR knockout pooled library (GeCKO v2) screens were performed as described previously [45], and lentiviral viruses were purchased from a local company (Azenta, USA). Approximately 1.2 × 10^8^ cells were infected with the mouse CRISPR knockout pooled library at a multiplicity of infection (MOI) < 0.3 (500× coverage). After 48 h of incubation, the cells were cultured for 5 days in 2iL medium supplemented with 1 µg/mL puromycin. Surviving cells were expanded and harvested as the control library. The mutant cells were further cultured for 28 days to reach at least 3.5 × 10^7^ cells. Following the 28‐day culture, the cells were collected and costained with 5 µg/mL Hoechst 33342 and 25 nm MitoTracker for 20 min at 37°C. The top 5% and bottom 5% of MitoTracker‐stained cells in the G0/G1 cell cycle phase were collected, followed by DNA extraction, sgRNA amplification, and sequencing as previously reported [46]. The sequencing reads were aligned and analyzed using MAGeCK [47] and MAGeCKFlute [48]. The sequences of the primers used for amplification are listed in Data S1.

### 4.13 Analysis of RNA‐seq Data

Total RNA was extracted and sequenced by a company (Azenta, USA) to generate 150‐bp paired‐end (PE150) raw reads. Raw RNA‑seq reads were trimmed and filtered using fastp (v1.0.1). Clean reads were mapped to the mouse reference genome (GRCm39) using HISAT2 (v2.2.1). Gene read counts were quantified and exported using featureCounts from the Subread package (v2.0.6) for downstream analysis. Differentially expressed gene (DEG) analysis was performed using DESeq2 (v1.46.0). All downstream RNA‑seq analyses were carried out in R (v 4.4.1). Heatmaps were generated using the R packages pheatmap (v1.0.13) and ClusterGVis (v0.1.2) [49].

### 4.14 Statistical Analysis

All the experiments in this study were performed with three independent biological replicates. No data transformation or normalization was applied unless otherwise specified. All continuous variables are presented as the mean ± standard deviation (SD), unless otherwise stated in the corresponding figure legends. The sample size (*n*) for each analysis represents the number of independent biological replicates, as specified in each figure legend. Specific statistical tests for each experiment are detailed in the corresponding figures and figure legends. All the statistical analyses were performed using GraphPad Prism 10.4 software. For comparisons across multiple groups, one‐way or two‐way ANOVA followed by Tukey's post hoc test was used. For comparisons between two independent groups, an unpaired two‐tailed Student's *t* test was applied. The significance threshold (α value) was set to 0.05. Statistical significance is denoted as follows: *ns*, not significant; ^*^ *P* < 0.05; ^**^ *P* < 0.01; ^***^ *P* < 0.001; ^****^ *P* < 0.0001.

## Author Contributions

L.S., C.T., and Q.G. conceptualized the idea and designed this study. L.S. and G.Q. supervised the study. Y.F., W.Z., Y‐F.Z., Y.H., and Y.D. performed most of the experiments. Y‐D.Z. and C.Y. contributed to the cell culture experiments. S.S. and X.S. were involved in the molecular biology experiments. Y.F., W.Z., and Y.D. analyzed the bioinformatics data. Y.F., W.Z., and L.S. wrote the manuscript draft. L.S. confirmed the final version.

## Conflicts of Interest

The authors declare no conflicts of interest.

## Supporting information




**Supporting File 1**: advs75425‐sup‐0001‐SuppMat.docx.

**Supporting File 2**: advs75425‐sup‐0002‐Data.xlsx.

**Supporting File 3**: advs75425‐sup‐0003‐Data1.xlsx.

## Data Availability

The datasets generated in this study have been deposited in the China National Center for Bioinformation (CNCB; https://www.cncb.ac.cn/) under accession number CRA040645.
