# Supplementary material for: Metabolic Imbalance Triggers Adaptive Remodeling to Accelerate Diploidization in Murine Haploid Embryonic Stem Cells
Source: Adv Sci (Weinh). 2026 Apr 22;13(40):e22570. doi: 10.1002/advs.202522570 (PMC13335663; doi:10.1002/advs.202522570)
Supplement: Supplementary file 1 — Supporting File 1: advs75425‐sup‐0001‐SuppMat.docx. [file ADVS-13-e22570-s003.docx]

**Title:** Metabolic Imbalance Triggers Adaptive Remodeling to Accelerate Diploidization in Murine Haploid Embryonic Stem Cells

**Running title:** Stabilizing Haploidy *via* Metabolic Regulation

**Authors and affiliations:**

Yi Fu^1,4^, Wenhao Zhang^1,4^, Yifan Zhang^1,4^, Yu He^2,4^, Yi Du^2^, Yiding Zhao^1^, Chunmeng Yao^1^, Shengyi Sun^1^, Xiaoyan Sheng^1^, Qian Gao^3*^, Chao Tong^2*^, and Ling Shuai^1,2*^

1 State Key Laboratory of Medicinal Chemical Biology, College of Pharmacy, Nankai University Animal Resources Center and Reproductive Regulation and Institute of Transplantation Medicine, Nankai University, Tianjin 300350, China.

2 Department of Neonatology, Children’s Hospital of Chongqing Medical University, National Clinical Research Center for Child Health and Disorders, Ministry of Education Key Laboratory of Child Development and Disorders, China International Science and Technology Cooperation Base of Child Development and Critical Disorders, Chongqing Key Laboratory of Child Rare Diseases in Infection and Immunity, Chongqing, 400014, China.

3 Department of Obstetrics and Gynecology, Chongqing Key Laboratory of Maternal and Fetal Medicine, The First Affiliated Hospital of Chongqing Medical University, Chongqing, 400016, China.

4 Co-first author

*** Correspondences:** gaoqian0171@163.com (Q.G.); chaotongcqmu@163.com (C.T.); lshuai@nankai.edu.cn (L.S.)

**Lead Contact:** lshuai@nankai.edu.cn (L.S.)


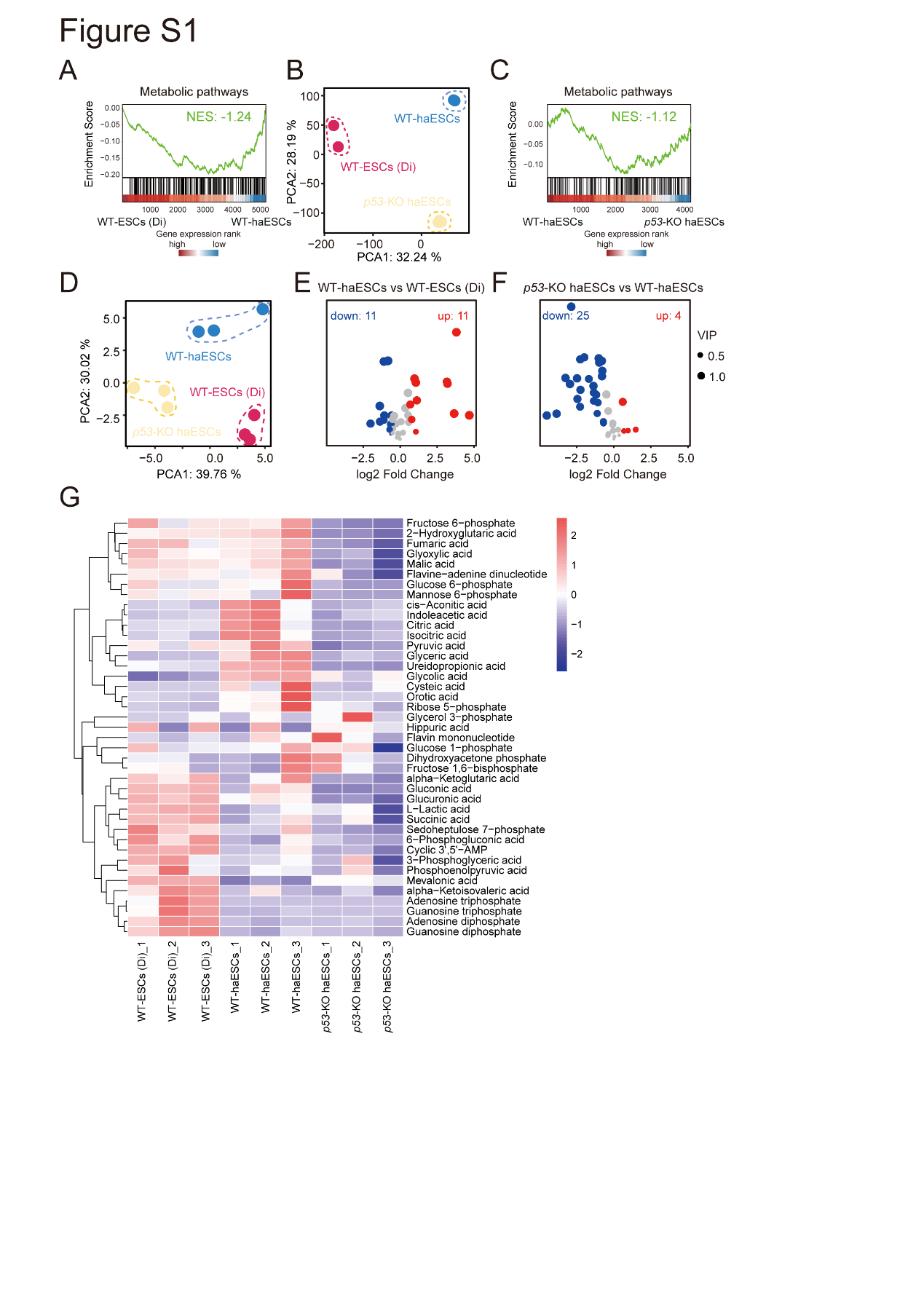


**Figure S1. Comparison of metabolic pathways between WT-ESCs (Di), WT-haESCs, and *p53*-KO-haESCs**

1. GSEA of WT-haESCs compared with WT-ESCs (Di) in Metabolic pathways.
2. Principal component analysis (PCA) of RNA-seq analysis between WT-ESCs (Di), WT-haESCs, and *p53*-KO haESCs (*n* = 2).
3. GSEA of *p53*-KO haESCs compared with WT-haESCs in Metabolic pathways.
4. PCA of metabolome analysis between WT-ESCs (Di), WT-haESCs, and *p53*-KO haESCs (*n* = 3).
5. Volcano plot analysis of metabolome analysis of WT-haESCs vs WT-ESCs (Di).
6. Volcano plot analysis of metabolome analysis of *p53*-KO haESCs vs WT-haESCs.
7. Heatmap result showing the abundance of metabolite in WT-ESCs (Di), WT-haESCs, and *p53*-KO haESCs (*n* = 3).


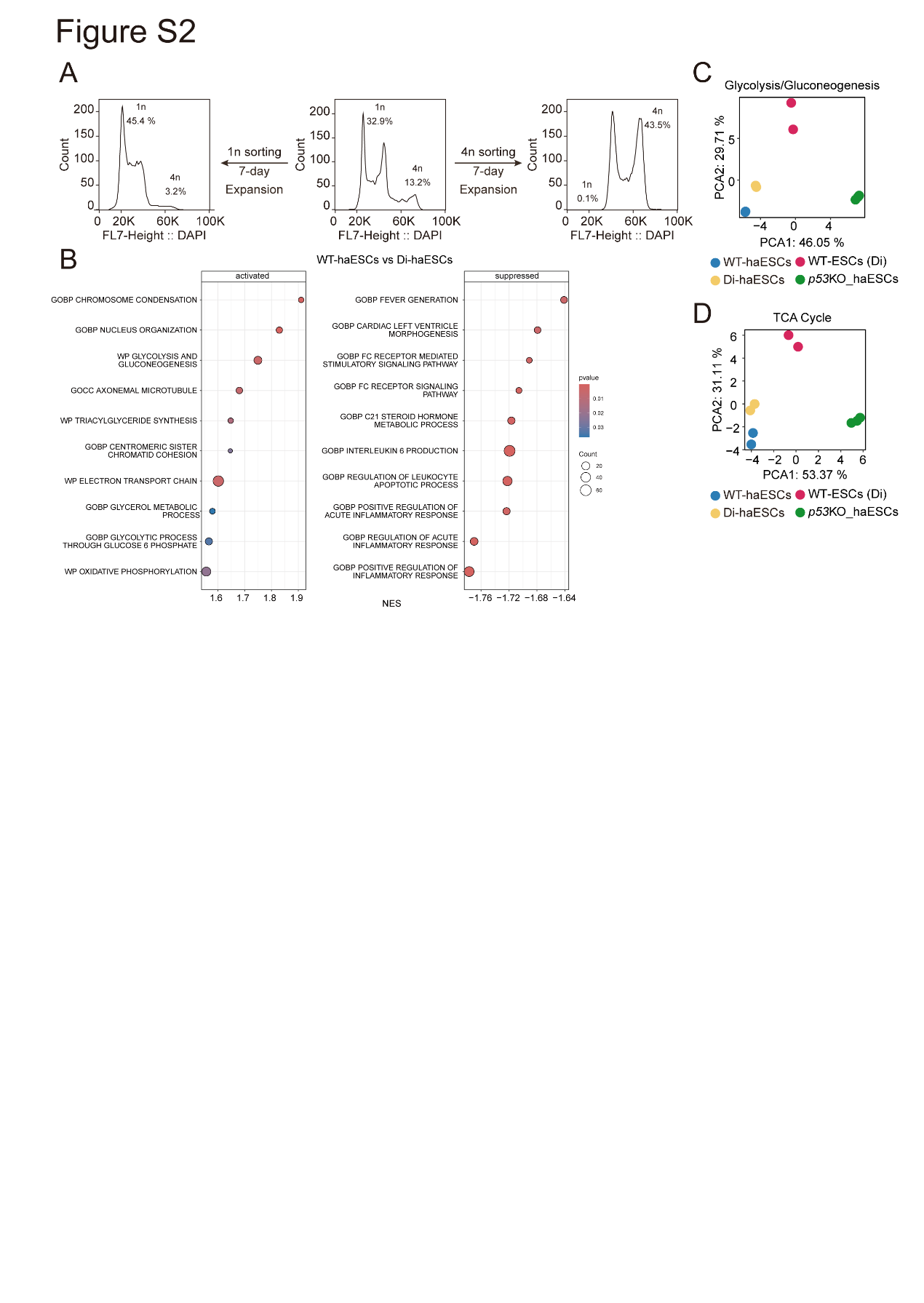


**Figure S2.** **Central carbon metabolism remodeling in the process of self-diploidization of WT-haESCs**

1. Representative flow cytometry analysis of DNA content. Left panel: ploidy distribution of the sorted 1n population after 7-day expansion, with the percentage of 1n peak and 4n peak indicated. Mid panel: ploidy distribution of unsorted WT-haESCs, with the percentage of 1n peak and 4n peak indicated. Right panel: ploidy distribution of the sorted 4n population after 7-day expansion, with the percentage of 1n peak and 4n peak indicated.
2. GSEA of WT-haESCs compared with Di-haESCs. Left panel: pathways activated in WT-haESCs compared with Di-haESCs (NES > 0); right panel: pathways suppressed in WT-haESCs compared with Di-haESCs (NES < 0).
3. Metabolic PCA of the Glycolysis/Gluconeogenesis pathway in WT-haESCs, Di-haESCs, WT-ESCs (Di), and *p53*KO-haESCs (*n* = 2, 2, 2, and 3). The variance explained by PCA1 and PCA2 is indicated.
4. Metabolic PCA of the TCA Cycle pathway in WT-haESCs, Di-haESCs, WT-ESCs (Di), and *p53*KO-haESCs (*n* = 2, 2, 2, and 3). The variance explained by PCA1 and PCA2 is indicated.


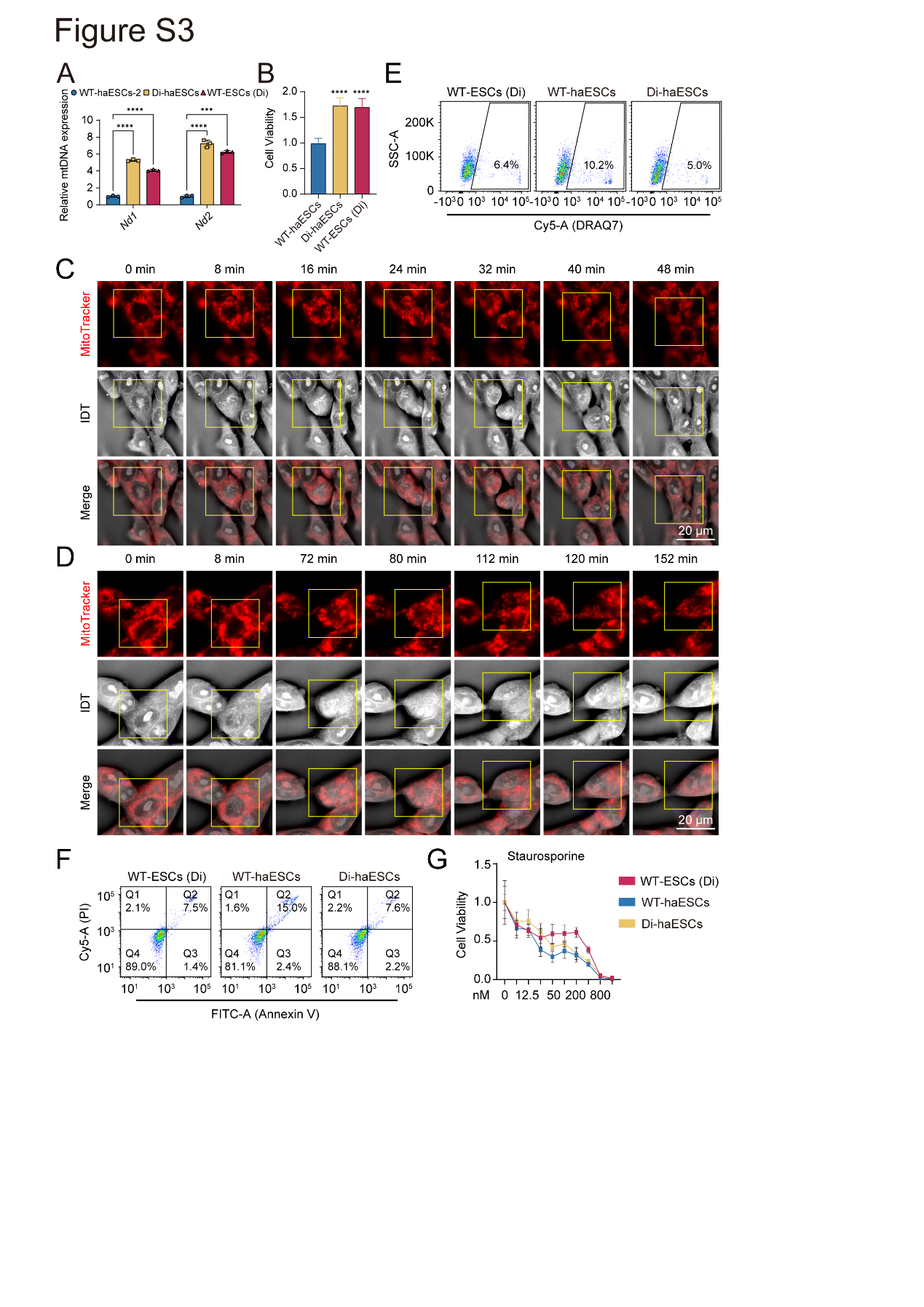


**Figure S3. Mitochondrial differences between WT-haESCs and Di-haESCs**

1. Quantification of relative mtDNA abundance in WT-haESCs-2, Di-haESCs-2, and WT-ESCs (Di). (*n* = 3, mean ± s.d.). Statistical significance was determined by two-way repeated-measures ANOVA followed by Tukey’s post-hoc test. ****P* < 0.001; *****P* < 0.0001.
2. CCK-8 assay results with WT-haESCs, Di-haESCs, and WT-ESCs (Di) after 2-day culture (*n* = 6, mean ± s.d.). Statistical significance was determined by one-way ANOVA with Tukey’s post-hoc test. *****P* < 0.0001.
3. Real-time imaging of normal dividing of WT-haESCs with MitoTracker staining. The yellow squares show the dividing cells. IDT, Intensity diffraction tomography.
4. Real-time imaging of abnormal dividing of WT-haESCs with MitoTracker staining. The yellow squares show the dividing cells. IDT, Intensity diffraction tomography.
5. The representative flow cytometry analysis of DRAQ7 in WT-ESCs (Di), WT-haESCs, and Di-haESCs.
6. The representative flow cytometry analysis of Annexin V/PI in WT-ESCs (Di), WT-haESCs, and Di-haESCs.
7. The growth curves of WT-ESCs (Di), WT-haESCs, and Di-haESCs in the presence of additional staurosporine using CCK8 assay (*n* = 6, mean ± s.d.).


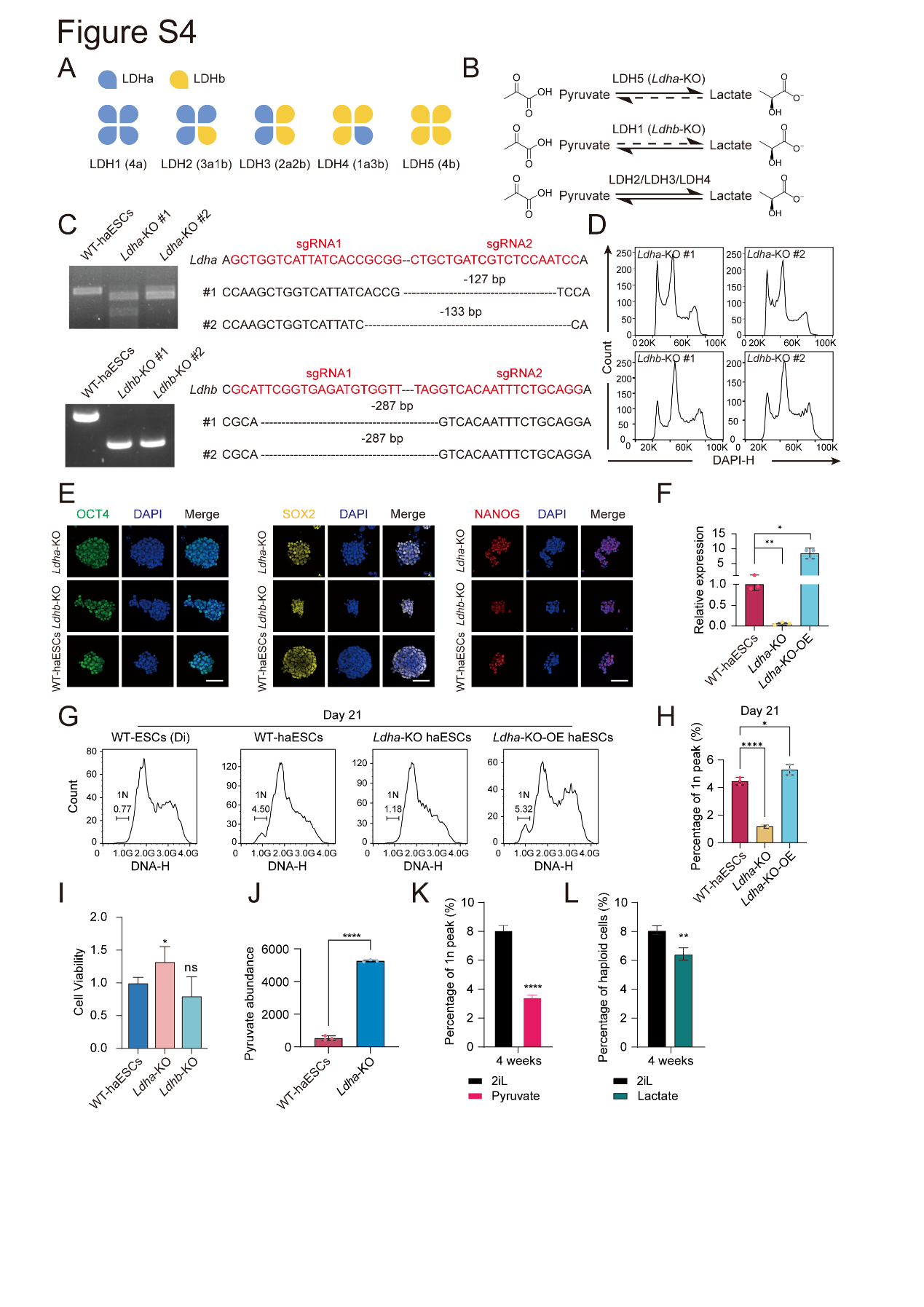


**Figure S4. Accelerated diploidization through *Ldha*-KO or addition of pyruvate**

1. Schematic diagram of the composition of LDH.
2. Schematic diagram showing the mutual conversion between lactic acid and pyruvic acid.
3. Genotype identification of *Ldha* and *Ldhb* gene knockout.
4. Flow cytometric analysis of DNA content in *Ldha*-KO and *Ldhb*-KO haESCs.
5. Representative immunofluorescence staining of WT-haESCs, *Ldha*-KO haESCs, and *Ldhb*-KO haESCs. Scale bar, 100 μm.
6. Quantification of relative *Ldha* mRNA expression in control (Ctrl), *Ldha*-KO haESCs, and *Ldha*-KO-OE haESCs (*n* = 3, mean ± s.d.). Statistical significance was determined by Brown-Forsythe and Welch ANOVA (to correct for heteroscedasticity), followed by Games-Howell post hoc test for pairwise comparisons between the control group and treatment groups. **P* < 0.05; ***P* < 0.01.
7. Representative flow cytometry analysis of DNA content in WT-ESCs (Di), WT-haESCs, *Ldha*-KO haESCs, and *Ldha*-KO-OE haESCs at day 21. The percentage of 1n peak is indicated in each panel.
8. Quantification of the percentage of 1n peak cells in control, *Ldha*-KO haESCs, and *Ldha*-KO-OE haESCs at day 21 (*n* = 3, mean ± s.d.). Statistical significance was determined by one-way ANOVA with Tukey’s post-hoc test. **P* < 0.05; *****P* < 0.0001.
9. CCK-8 assay results with WT-haESCs, *Ldha*-KO haESCs, and *Ldhb*-KO haESCs after 2-day culture (*n* = 4, mean ± s.d.). Statistical significance was determined by one-way ANOVA with Tukey’s post-hoc test. *ns*, not significant; **P* < 0.05.
10. Quantification of intracellular pyruvate abundance in WT-haESCs and *Ldha*-KO haESCs (*n* = 3, mean ± s.d.). Statistical significance was determined by unpaired two-tailed Student’s t-test. *****P* < 0.0001.
11. Quantification of flow cytometric analysis of DNA content in WT-haESCs cultured in 2iL with or without pyruvate after 4-week culture (*n* = 3, mean ± s.d.). Statistical significance was determined by unpaired two-tailed Student’s t-test. *****P* < 0.0001.
12. Quantification of flow cytometric analysis of DNA content in WT-haESCs cultured in 2iL with or without lactate after 4-week culture (*n* = 3, mean ± s.d.). Statistical significance was determined by unpaired two-tailed Student’s t-test. ***P* < 0.01.


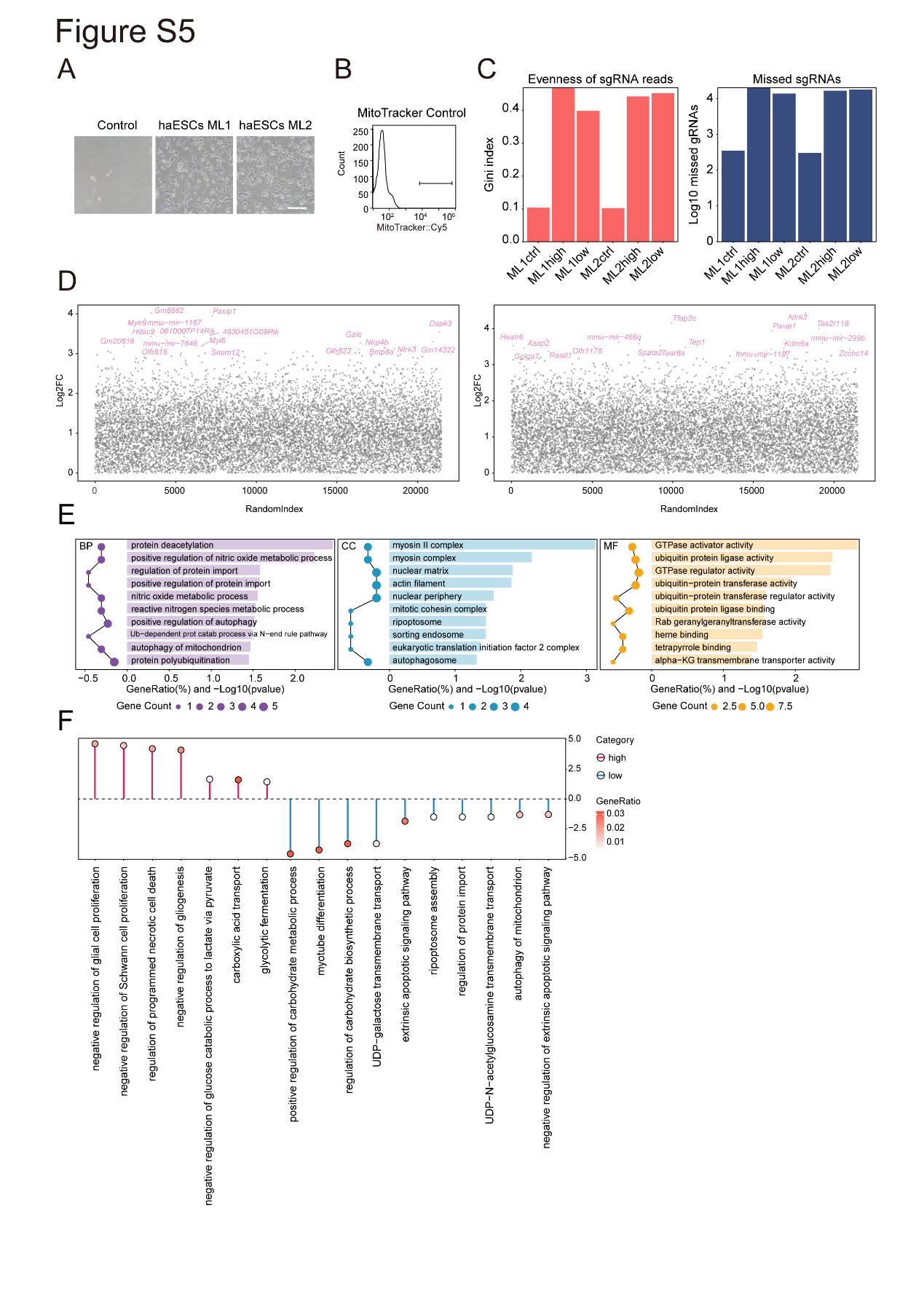


**Figure S5. Identifying key genes of mitochondrial quality-control during the diploidization process *via* genome-wide screening of haESCs**

1. Representative BF images of WT-haESCs, haESCs mutate library (ML) 1 and ML2. Scale bars, 100 μm.
2. The negative control of MitoTracker in Figure 5B.
3. Quality control of CRISPR screening.
4. Plot displays Log2 foldchange from MAGeCK analysis of gene counts between MitoTracker^high^ (right)/ MitoTracker^low^ (left) treated cells.
5. GO analysis results of significantly enriched genes from Fig. 5c.
6. GO analysis results of significantly enriched genes in MitoTracker^high^ and MitoTracker^low^ populations, respectively.


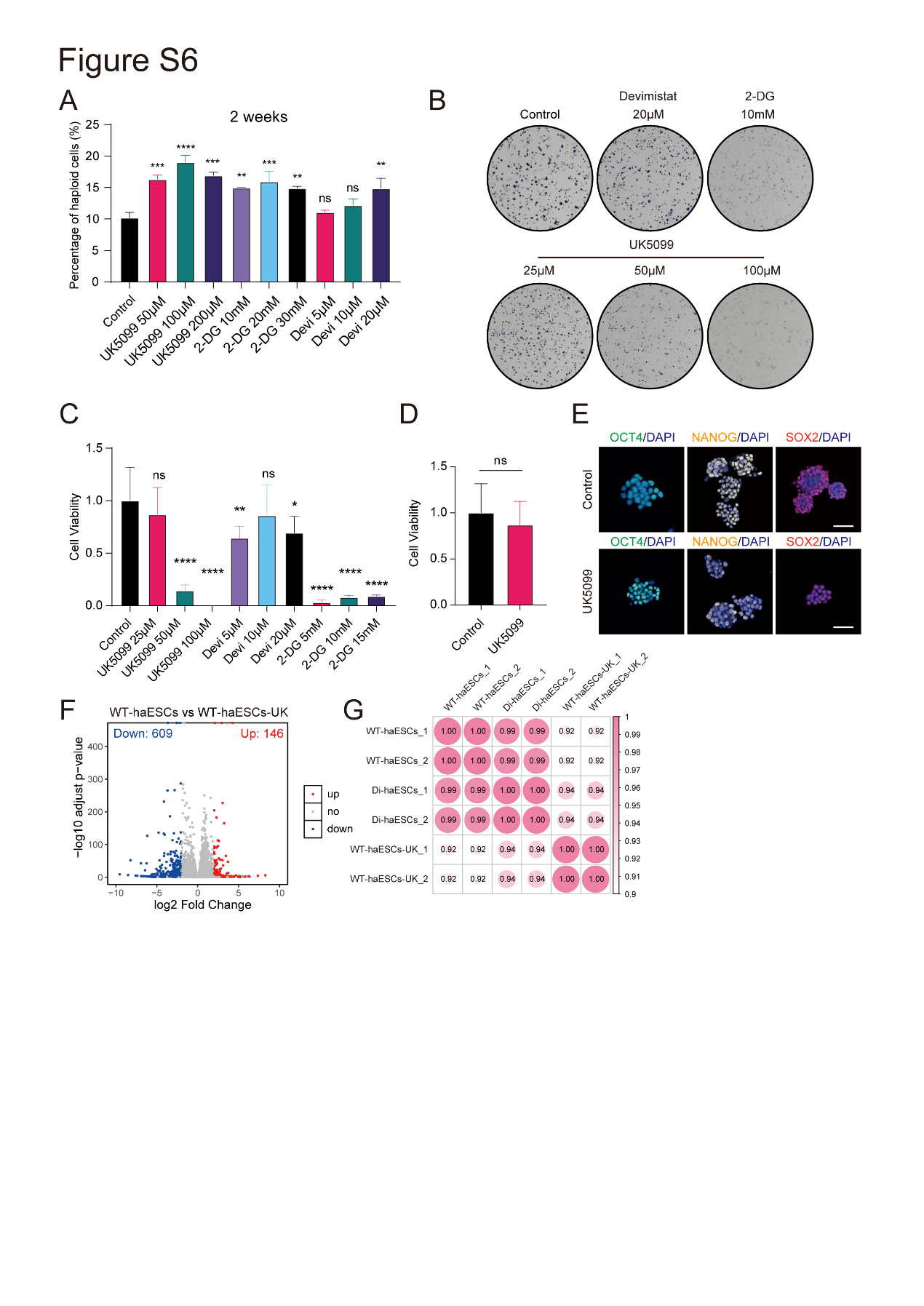


**Figure S6. Inhibition of mitochondrial pyruvate uptake promotes haploidy maintenance**

1. Percentage of 1n peak of WT-haESCs cultured in 2iL medium, 2iL medium adding UK5099, 2-DG or devimistat after 2-week culture (*n* = 2, mean ± s.d.). Statistical significance was determined by one-way ANOVA with Tukey’s post-hoc test. *ns*, not significant; ***P* < 0.01; ****P* < 0.001; *****P* < 0.0001.
2. Crystal violet staining of WT-haESCs cultured in 2iL medium, 2iL medium adding UK5099, 2-DG or devimistat after 2-day culture.
3. CCK-8 assay results with WT-haESCs cultured in 2iL medium, 2iL medium adding UK5099, 2-DG or devimistat after 2-day culture (*n* = 6, mean ± s.d.). Statistical significance was determined by one-way ANOVA with Tukey’s post-hoc test. *ns*, not significant; **P* < 0.05; ***P* < 0.01; *****P* < 0.0001.
4. CCK-8 assay result with WT-haESCs cultured in 2iL medium with or without UK5099 in 2-day culture (*n* = 6, mean ± s.d.). Statistical significance was determined by unpaired two-tailed Student’s t-test. *ns*, not significant.
5. Representative immunofluorescence staining of WT-haESCs cultured in 2iL medium with or without UK5099. Scale bar, 100 μm.
6. Volcano plot analysis showing the different expression genes between WT-haESCs cultured in 2iL medium with or without UK5099.
7. Correlation analysis of RNA-seq among WT-haESCs, Di-haESCs, and WT-haESCs cultured in UK5099 (*n* = 2).


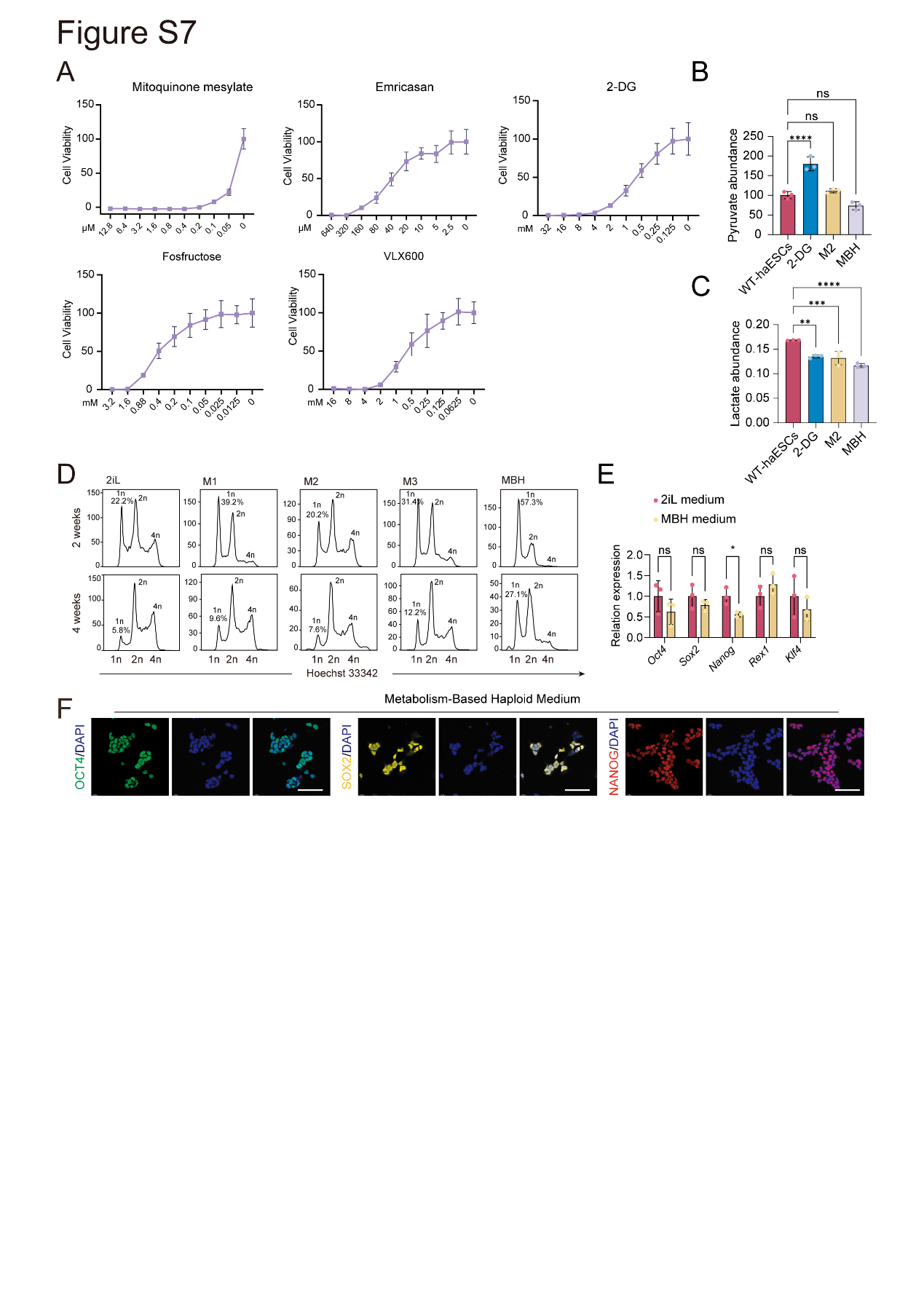


**Figure S7. An optimized culture medium for haploidy maintenance**

1. Cell viability assay of WT-haESCs treated with gradient concentrations of emricasan, mitoquinone mesylate, 2-DG, fosfructose, and VLX600 (*n* = 6, mean ± s.d.). Final working concentrations are indicated by dashed lines.
2. Quantification of intracellular pyruvate abundance in WT-haESCs, 2-DG-treated haESCs, M2 medium-cultured haESCs and MBH medium-cultured haESCs (*n* = 3, mean ± s.d.). Statistical significance was determined by one-way ANOVA with Tukey’s post-hoc test. *ns*, not significant; *****P* < 0.0001.
3. Quantification of intracellular lactate abundance in WT-haESCs, 2-DG-treated haESCs, M2 medium-cultured haESCs, and MBH medium-cultured haESCs (*n* = 3, mean ± s.d.). Statistical significance was determined by one-way ANOVA with Tukey’s post-hoc test. ***P* < 0.01; ****P* < 0.001; *****P* < 0.0001.
4. Representative flow cytometry analysis of DNA content in WT-haESCs cultured in 2iL medium with different modules for 2 and 4 weeks.
5. Quantification of relative mRNA expression of pluripotency markers (*Oct4*, *Sox2*, *Nanog*, *Rex1*, *Klf4*) in 2iL medium and MBH medium-cultured haESCs (*n* = 3, mean ± s.d.). Statistical significance was determined by unpaired two-tailed Student’s t-test. *ns*, not significant; **P* < 0.05.
6. Representative immunofluorescent staining of pluripotency markers OCT4, SOX2 and NANOG in 2iL medium and MBH medium-cultured haESCs. Nuclei were stained with DAPI. Scale bar, 100 μm.
